# Supplementary material for: Fermi polarons under strain-induced pseudomagnetic fields
Source: Nat Commun. 2025 Nov 20;16:10232. doi: 10.1038/s41467-025-66192-y (PMC12635052; doi:10.1038/s41467-025-66192-y)
Supplement: Supplementary file 1 — Supplementary Information [file 41467_2025_66192_MOESM1_ESM.pdf]

Supplementary Information:
Fermi polarons under strain-induced pseudomagnetic fields

Denis Yagodkin[1], Kenneth Burfeindt[1], Zakhar A. Iakovlev[3], Abhijeet M. Kumar[1], Adrián Dewambrechies[1], Oguzhan Yücel[1], Bianca Höfer[1], Cornelius Gahl[1], Mikhail M. Glazov[3], and Kirill I. Bolotin[1,2]

[1]*Department of Physics, Freie Universität Berlin, Arnimallee 14, 14195 Berlin, Germany*
[2]*Halle-Berlin-Regensburg Cluster of Excellence CCE @ Freie Universität Berlin, Berlin, Germany*
[3]*Ioffe Institute, Polytechnicheskaya 26, 194021, St. Petersburg, Russia*

2025-10-16

Contents

Contents **1**

S1 Theory of pseudospin in pseudomagnetic field **3**
S1.1 Pseudospin relaxation 3
S1.2 Energy relaxation and pseudomagnetic field induced polarization 5
S1.3 Photoluminescence spectra of excitons in pseudomagnetic field 9

S2 Calibration of strain **12**

S3 Analysis of Pseudospin Time Constants **15**

S4 Estimate of the exciton LT splitting **19**

S5 Calculation of Fermi energy **20**

S6 Evaluation of Fermi polaron pseudo-Zeeman splitting **21**

S7 Simulation of pseudo-Zeeman splitting at large pseudomagnetic fields **22**

References **36**

List of Figures

S1 Strain distribution 24

S2 Extended analysis of WSe_2 device 25
S3 Extended analysis of $MoSe_2$ devices 26
S4 Exciton Pseudo-Zeeman splitting measured in reflectivity 27
S5 $MoSe_2$ under biaxial vs uniaxial strain. 28
S6 Emergence of orthogonal pseudospin component in pseudomagnetic field 29
S7 Pseudo-Larmor effect in WSe_2 at various polarizations of excitation 30
S8 Dependence of S_z of X_0, X^-, XX, X_d^+ on pseudomagnetic field 31
S9 Strain calibration 32
S10 Quantification of uniaxial strain using polarization-resolved Raman spectroscopy. . . 33
S11 The effect of pseudomagnetic field on relaxation time. 34
S12 Zeeman splitting at high pseudomagnetic field 35

S1 Theory of pseudospin in pseudomagnetic field

S1.1 Pseudospin relaxation

We describe exciton pseudospin dynamics within the framework of the kinetic equation for the 2×2 density matrix [1, 2, 3]

$$\rho_{\boldsymbol{k}} = n_{\boldsymbol{k}} \hat{\sigma}_0 + (\boldsymbol{s}_{\boldsymbol{k}} \cdot \hat{\boldsymbol{\sigma}}) \,.$$

Here $\boldsymbol{k}$ is the exciton in plane wavevector, $n_{\boldsymbol{k}} = \mathrm{Sp}\{\rho_{\boldsymbol{k}}\}/2$ is the pseudospin averaged occupancy of the orbital state with the wavevector $\boldsymbol{k}$, $\boldsymbol{s}_{\boldsymbol{k}} = \mathrm{Sp}\{\rho_{\boldsymbol{k}} \hat{\boldsymbol{\sigma}}\}/2$ is the average pseudospin in this orbital state; $\hat{\sigma}_0$ is the unit 2×2 matrix and $\hat{\boldsymbol{\sigma}} = (\hat{\sigma}_x, \hat{\sigma}_y, \hat{\sigma}_z)$ is the vector composed of the Pauli matrices. The kinetic equation for the vector $\boldsymbol{s}_{\boldsymbol{k}}$ reads (the time argument is omitted for brevity)

$$\frac{\partial \boldsymbol{s}_{\boldsymbol{k}}}{\partial t} + \boldsymbol{s}_{\boldsymbol{k}} \times \boldsymbol{\Omega}_{\boldsymbol{k}} + \frac{\boldsymbol{s}_{\boldsymbol{k}}}{\tau} + \mathrm{St}\{\boldsymbol{s}_{\boldsymbol{k}}\} = \boldsymbol{g}_k, \tag{S1}$$

where τ is the exciton lifetime, $\boldsymbol{g}_k$ is the pseudospin generation rate (assumed to be independent of orientation of $\boldsymbol{k}$),

$$\mathrm{St}\{\boldsymbol{s}_{\boldsymbol{k}}\} = \frac{\boldsymbol{s}_{\boldsymbol{k}} - \langle \boldsymbol{s}_{\boldsymbol{k}} \rangle}{\tau_{sc}}, \quad \langle \boldsymbol{s}_{\boldsymbol{k}} \rangle = \int_0^{2\pi} \frac{d\varphi_{\boldsymbol{k}}}{2\pi} \boldsymbol{s}_{\boldsymbol{k}},$$

is the collision integral described, for simplicity, by a single relaxation time τ_{sc} responsible for isotropization of the non-equilibrium distribution function, $\varphi_{\boldsymbol{k}}$ is the polar angle of the wavevector $\boldsymbol{k}$, and $\boldsymbol{\Omega}_{\boldsymbol{k}}$ is the effective pseudospin precession frequency (pseudomagnetic field) that consists of two contributions

$$\boldsymbol{\Omega}_{\boldsymbol{k}} = \boldsymbol{\Omega} + \boldsymbol{\Omega}_{\boldsymbol{k}}^{LT}, \tag{S2}$$

with $\boldsymbol{\Omega} \parallel x$ being the momentum-independent contribution related to the anisotropic strain and $\boldsymbol{\Omega}_{\boldsymbol{k}}^{LT}$ is the exciton longitudinal-transverse splitting. The latter can be recast as

$$\boldsymbol{\Omega}_{\boldsymbol{k}}^{LT} = \Omega_k^{LT} \times (\cos 2\varphi_{\boldsymbol{k}}, \sin 2\varphi_{\boldsymbol{k}}, 0),$$

with $\Omega_k^{LT} \propto k$, see Refs. [4, 5, 6, 3] for details. Equation (S1) disregards the (slow) energy relaxation processes, they are taken into account in the next subsection S1.2.

Under assumptions of $\Omega_{\boldsymbol{k}}^{LT} \tau_{sc} \ll 1$ and $\tau_{sc} \ll \tau$ Eq. (S1) can be solved by iterations in the small parameter $\Omega_{\boldsymbol{k}}^{LT} \tau_{sc}$ with the resulting equation for the angular average pseudospin, $\langle \boldsymbol{s}_{\boldsymbol{k}} \rangle$, in the form

$$-\mathrm{i}\omega \langle \boldsymbol{s}_{\boldsymbol{k}} \rangle_\omega + [1 - \Xi(\omega)] \langle \boldsymbol{s}_{\boldsymbol{k}} \rangle_\omega \times \boldsymbol{\Omega} + \frac{\langle \boldsymbol{s}_{\boldsymbol{k}} \rangle_\omega}{\tau} + \hat{\Gamma}(\omega) \langle \boldsymbol{s}_{\boldsymbol{k}} \rangle_\omega = \boldsymbol{g}_{k,\omega}. \tag{S3}$$

Here the subscript ω describes the corresponding Fourier component

$$\langle \boldsymbol{s}_{\boldsymbol{k}} \rangle_\omega = \int_{-\infty}^{\infty} dt e^{\mathrm{i}\omega t} \langle \boldsymbol{s}_{\boldsymbol{k}}(t) \rangle, \quad \boldsymbol{g}_{k,\omega} = \int_{-\infty}^{\infty} dt e^{\mathrm{i}\omega t} \boldsymbol{g}_k(t),$$

and $\hat{\Gamma}(\omega)$ is the effective relaxation rate tensor, and $\Xi(\omega)\boldsymbol{\Omega}$ is effective renormalization of pseudomagnetic field. The components of the tensor $\hat{\Gamma}(\omega)$ and scalar $\Xi(\omega)$ depend on the frequency and are, in general, complex. Introducing

$$\tau_\omega = \frac{\tau_{sc}}{1 - \mathrm{i}\omega\tau_{sc}},$$

we express these parameters as

$$\Xi(\omega) = \frac{(\Omega_k^{LT}\tau_\omega)^2}{2[1+(\Omega\tau_\omega)^2]}, \tag{S4}$$

and

$$\Gamma_{xx}(\omega) = \Gamma_{yy}(\omega) = \frac{(\Omega_k^{LT})^2\tau_\omega}{2[1+(\Omega\tau_\omega)^2]}, \tag{S5a}$$

$$\Gamma_{zz}(\omega) = \Gamma_{xx}(\omega) + \frac{(\Omega_k^{LT})^2\tau_\omega}{2}, \tag{S5b}$$

Equation (S3) is the analogue of the pseudospin Bloch equation [Eq. (2) of the main text] microscopically derived for the relevant situation where the pseudospin relaxation and dynamics is controlled by the longitudinal-transverse splitting of excitons $\Omega_{\boldsymbol{k}}^{\rm LT}$. The principal issue here is that both effective pseudospin precession frequency $\boldsymbol{\Omega}[1-\Xi(\omega)]$ and the relaxation rates $\hat{\Gamma}(\omega)$ are frequency dependent. It provides a memory effect in pseudospin dynamics and makes it, generally, more complicated compared to that described by the classical Bloch equation. For example, the expressions for Γ_{xx}, Γ_{yy}, and Γ_{zz} at $\omega = \Omega$ are presented in Ref. [3], Eqs. (10); while the experimentally relevant case of $\omega = 0$ is studied below.

In accordance with experimental situation we focus on the steady-state excitation where $g_k = \mathrm{const}(t)$. Setting $\omega = 0$ in Eqs. (S5) renders both the scalar $\Xi(0)$ and the tensor $\hat{\Gamma}(0)$ real [cf. Ref. [7]] and we obtain

$$\Gamma_{xx} = \frac{\Gamma_s}{1+(\Omega\tau_{sc})^2}, \tag{S6a}$$

$$\Gamma_{yy} = \frac{\Gamma_s}{1+(\Omega\tau_{sc})^2}, \tag{S6b}$$

$$\Gamma_{zz} = \frac{\Gamma_s}{1+(\Omega\tau_{sc})^2} + \Gamma_s, \tag{S6c}$$

where [cf. Ref. [8]]

$$\Gamma_s = \frac{(\Omega_k^{LT})^2\tau_{sc}}{2}. \tag{S7}$$

The pseudomagnetic field correction $\Xi(0)$ can be neglected for steady-state excitation.

Equations above were derived at $\Omega_k^{LT}\tau_{sc} \ll 1$. At arbitrary value of the product $\Omega_k^{LT}\tau_{sc}$ we obtain at $\omega = 0$

$$\Gamma_{xx} = \Gamma_{yy} = \frac{\left(\Omega_k^{LT}\right)^2\tau_{sc}}{1+(\Omega\tau_{sc})^2 + \sqrt{\left[1+(\Omega\tau_{sc})^2+\left(\Omega_k^{LT}\tau_{sc}\right)^2\right]^2 - \left(2\Omega\Omega_k^{LT}\tau_{sc}^2\right)^2}}, \tag{S8a}$$

$$\Gamma_{zz} = \Gamma_{xx} + \frac{\sqrt{\left[1+(\Omega\tau_{sc})^2+\left(\Omega_k^{LT}\tau_{sc}\right)^2\right]^2 - \left(2\Omega\Omega_k^{LT}\tau_{sc}^2\right)^2}\left(\Omega_k^{LT}\right)^2\tau_{sc}}{1+(\Omega\tau_{sc})^2 + \sqrt{\left[1+(\Omega\tau_{sc})^2+\left(\Omega_k^{LT}\tau_{sc}\right)^2\right]^2 - \left(2\Omega\Omega_k^{LT}\tau_{sc}^2\right)^2}}, \tag{S8b}$$

$$\Xi(0) = \Gamma_{xx}\tau_{sc}. \tag{S8c}$$

The steady-state solution of Eq. (S3) can be written in the cases of interest as follows.

1. *Linearly polarized excitation along in the principal axes of deformation,* $\boldsymbol{g}_k \parallel x$

$$\langle s_{\boldsymbol{k},x}\rangle = \frac{g_{k,x}}{\tilde{\Gamma}_{xx}}, \quad \langle s_{\boldsymbol{k},y}\rangle = \langle s_{\boldsymbol{k},z}\rangle = 0. \tag{S9a}$$

2. *Linearly polarized excitation at* 45° *to the prinicpal axes of deformation,* $\boldsymbol{g}_k \parallel y$

$$\langle \boldsymbol{s_k}\rangle = \frac{\tilde{\Gamma}_{zz}\boldsymbol{g}_k}{\tilde{\Omega}^2 + \tilde{\Gamma}_{yy}\tilde{\Gamma}_{zz}} + \frac{\tilde{\boldsymbol{\Omega}} \times \boldsymbol{g}_k}{\tilde{\Omega}^2 + \tilde{\Gamma}_{yy}\tilde{\Gamma}_{zz}}. \tag{S9b}$$

3. *Circularly polarized excitation,* $\boldsymbol{g}_k \parallel z$

$$\langle \boldsymbol{s_k}\rangle = \frac{\tilde{\Gamma}_{yy}\boldsymbol{g}_k}{\tilde{\Omega}^2 + \tilde{\Gamma}_{yy}\tilde{\Gamma}_{zz}} + \frac{\tilde{\boldsymbol{\Omega}} \times \boldsymbol{g}_k}{\tilde{\Omega}^2 + \tilde{\Gamma}_{yy}\tilde{\Gamma}_{zz}}. \tag{S9c}$$

Here

$$\tilde{\boldsymbol{\Omega}} = [1 - \Xi(0)]\boldsymbol{\Omega}$$

takes into account the effect of renormalization at $\Omega_k^{LT}\tau_{sc} \gtrsim 1$, the effect of the finite lifetime can be taken into account via renormalization

$$\tilde{\Gamma}_{\alpha\beta} = \Gamma_{\alpha\beta} + \delta_{\alpha\beta}\frac{1}{\tau}, \quad \alpha, \beta = x, y, z,$$

where $\delta_{\alpha\beta}$ is the Kronecker's δ-symbol.

S1.2 Energy relaxation and pseudomagnetic field induced polarization

In addition to the optically induced pseudospin polarization, the presence of the deformation-induced splitting results in the quasi-equilibrium, thermal, polarization of excitons.

To calculate the thermal quasi-equilibrium polarization of excitons we need to take into account both pseudospin and energy relaxation processes. To simplify the analysis we assume that the isotropization time of the exciton distribution function (τ_{sc}) is much shorter that both lifetime and energy relaxation time and consider only the "slow" processes. We are interested in the spin polarization along the direction of the pseudomagnetic field, thus, instead of the full 2×2 spin density matrix it is sufficient to consider only the occupancies of the excitons with the pseudospins parallel and antiparallel to the x-axis. We denote these states as $\uparrow$ and $\downarrow$ for brevity. The system of kinetic equations for the spin up ($\uparrow$) and spin down ($\downarrow$) excitons reads

$$\frac{f_\uparrow(\varepsilon)}{\tau} + \frac{f_\uparrow(\varepsilon) - f_\downarrow(\varepsilon)}{2\tau_s} + \frac{d}{d\varepsilon}\left[\hat{J} f_\uparrow(\varepsilon)\right] = \delta(\varepsilon - E_0)g_0, \tag{S10a}$$

$$\frac{f_\downarrow(\varepsilon)}{\tau} - \frac{f_\uparrow(\varepsilon) - f_\downarrow(\varepsilon)}{2\tau_s} + \frac{d}{d\varepsilon}\left[\hat{J} f_\downarrow(\varepsilon)\right] = \delta(\varepsilon - E_0)g_0. \tag{S10b}$$

Here, ε is the exciton energy, $f_\uparrow(\varepsilon), f_\downarrow(\varepsilon)$ are the distribution functions of excitons with spin up and down, respectively; $\tau_s(\varepsilon) = 1/\Gamma_{xx}(\varepsilon)$ is the spin relaxation time along the direction of the magnetic field. Note, that here we consider Γ_{xx} (not $\tilde{\Gamma}_{xx}$) as we take into account finite exciton

lifetime separately. The energy relaxation is described as the energy diffusion (cf. Ref. [9]). The energy flux operator is defined as

$$\hat{J} = D(\varepsilon)\left(\frac{1}{k_B T} + \frac{d}{d\varepsilon}\right), \tag{S11}$$

where $D(\varepsilon)$ is energy diffusion coefficient. The right hand sides of Eqs. (S10) correspond to the spin-isotropic pumping at the high energy $E_0 \gg k_B T$. In what follows, for simplicity, we disregard the energy dependence of the spin relaxation time replacing the relaxation time by its typical value $\tau_s(\varepsilon) \equiv \tau_s$.

We assume that the energy spans from $-\hbar\Omega/2$ to $+\infty$. The bottom of the spin up subband is, thus, at $+\hbar\Omega/2$ and the bottom of the spin down subband is at $-\hbar\Omega/2$. In the narrow energy band , $-\hbar\Omega/2 < \varepsilon < \hbar\Omega/2$, there exists only spin down states, and the kinetic equation simplifies to

$$\frac{f_\downarrow(\varepsilon)}{\tau} + \frac{d}{d\varepsilon}\left[\hat{J} f_\downarrow(\varepsilon)\right] = 0, \tag{S12}$$

while $f_\uparrow$ is irrelevant for this energy band. Here, we assume that the spin splitting is small in comparison with the temperature, $\hbar\Omega \ll k_B T$.

The boundary conditions are straightforwardly formulated in terms of fluxes. In particular, there is no current at the bottoms of subbands

$$\hat{J} f_\uparrow\left(\frac{\hbar\Omega}{2}\right) = 0, \qquad \hat{J} f_\downarrow\left(-\frac{\hbar\Omega}{2}\right) = 0. \tag{S13}$$

The exciton generation at $\varepsilon = E_0$ can also be recast as a boundary condition in the form

$$\hat{J} f_\uparrow(E_0) = \hat{J} f_\downarrow(E_0) = -g_0. \tag{S14}$$

It is convenient to rewrite the boundary conditions (S13) in somewhat different form. As we consider $\hbar\Omega \ll k_B T$, the distribution function $f_\downarrow(\varepsilon)$ can be taken as a constant in the range $-\hbar\Omega/2 < \varepsilon < \hbar\Omega/2$. After the integration of Eq. (S12) over energy ε from $-\hbar\Omega/2$ to $\hbar\Omega/2$, we express the energy flux at $\varepsilon = \hbar\Omega/2$ as

$$\hat{J} f_\downarrow\left(\frac{\hbar\Omega}{2}\right) = -\frac{\hbar\Omega}{\tau} f(0). \tag{S15}$$

Next, we rewrite the kinetic equations in terms of the exciton population distribution function

$$f(\varepsilon) = \frac{f_\uparrow(\varepsilon) + f_\downarrow(\varepsilon)}{2} = \langle n_{\boldsymbol{k}} \rangle \tag{S16}$$

and the exciton spin distribution function

$$s_x(\varepsilon) = \frac{f_\uparrow(\varepsilon) - f_\downarrow(\varepsilon)}{2} = \langle s_{\boldsymbol{k},x} \rangle. \tag{S17}$$

The kinetic equations take a form

$$\frac{f}{\tau} + \frac{d}{d\varepsilon}\left[\hat{J} f(\varepsilon)\right] = 0, \tag{S18a}$$

$$\frac{s_x}{\tau} + \frac{s_x}{\tau_s} + \frac{d}{d\varepsilon}\left[\hat{J} s_x(\varepsilon)\right] = 0, \tag{S18b}$$

with the boundary conditions

$$\hat{J} f(0) = -\hat{J} s_x(0) = -\frac{\hbar\Omega}{2\tau}\left[f(0) - s_x(0)\right], \tag{S19a}$$

$$\hat{J} f(E_0) = -g_0, \qquad \hat{J} s_x(E_0) = 0. \tag{S19b}$$

Note that for these equations it is already unimportant where the bottom of the band is set provided that $\hbar\Omega \ll k_B T$. Hereafter we set the bottom energy boundary to zero. For the same reason, the spin polarization $s_x(\varepsilon) \propto \hbar\Omega$ is small in comparison with $f(\varepsilon)$ and can be neglected in the boundary condition (S19a). Moreover, we can entirely omit the $\propto \hbar\Omega$ contribution in the boundary condition for $\hat{J} f(0)$ as we are working in the lowest order in pseudomagnetic field. Thus, the boundary conditions simplify to

$$\hat{J} f(0) = 0, \qquad \hat{J} s_x(0) = \frac{\hbar\Omega}{2\tau} f(0), \tag{S20a}$$

$$\hat{J} f(E_0) = -g_0, \qquad \hat{J} s_x(E_0) = 0, \tag{S20b}$$

and the kinetic equations separates: first, we can solve the kinetic equation for population $f(\varepsilon)$, and then substitute the results in boundary conditions for exciton spin $s_x(\varepsilon)$ and solve for $s_x(\varepsilon)$. Finally, we consider the lifetime sufficiently large, $D(\varepsilon)\tau \gg (k_B T)^2$, such that excitons manage to thermalize as

$$f(\varepsilon) = f_0 \exp\left(-\frac{\varepsilon}{k_B T}\right). \tag{S21}$$

Integrating Eq. (S18a) over ε, we get the obvious result, that the exciton generation rate is equal to their annihilation rate

$$-\hat{J} f(E_0) = g_0 = \frac{1}{\tau}\int_0^{E_0} f_0 \exp\left(-\frac{\varepsilon}{k_B T}\right) d\varepsilon = \frac{k_B T}{\tau} f(0). \tag{S22}$$

Similar integration of the kinetic equation (S18b) for spin gives

$$\left(\frac{1}{\tau} + \frac{1}{\tau_s}\right)\int_0^{E_0} s_x(\varepsilon) d\varepsilon = \hat{J} s_x(0) = \frac{\hbar\Omega}{2\tau} f(0). \tag{S23}$$

The total exciton pseudospin contains also the contribution from the $\downarrow$ particles with the energies $-\hbar\Omega/2 < \varepsilon < \hbar\Omega/2$:

$$\int_{-\hbar\Omega/2}^{\hbar\Omega/2} s_x(\varepsilon) d\varepsilon = -\frac{1}{2}\int_{-\hbar\Omega/2}^{\hbar\Omega/2} f_\downarrow(\varepsilon) d\varepsilon = -\hbar\Omega\frac{f(0)}{2}. \tag{S24}$$

Combining Eqs. (S23) and (S24) we obtain

$$\int_{-\hbar\Omega/2}^{E_0} s_x(\varepsilon) d\varepsilon = -\frac{\tau}{\tau + \tau_s}\frac{\hbar\Omega}{2} f(0) = -\frac{\tau}{\tau + \tau_s}\frac{\hbar\Omega}{2k_B T}\int_{-\hbar\Omega/2}^{E_0} f(\varepsilon) d\varepsilon. \tag{S25}$$

Normalizing exciton pseudospin to exciton occupancy we have finally

$$\boldsymbol{S}_0 = -\frac{\tau}{\tau+\tau_s}\frac{\hbar\boldsymbol{\Omega}}{2k_B T}. \tag{S26}$$

Equation (S26) shows that the pseudomagnetic field induced pseudospin polarization is controlled by the dynamical factor $\tau/(\tau+\tau_s)$ that describes the probability for exciton to flip its pseudospin during the lifetime. In the general case of arbitrary ratio of $\hbar\Omega$ and $k_B T$ we have for the induced spin polarization $\boldsymbol{S}_0$

$$\boldsymbol{S}_0 = -\frac{\tau}{\tau+\tau_s}\tanh\left(\frac{\hbar\Omega}{2k_B T}\right)\frac{\boldsymbol{\Omega}}{\Omega}. \tag{S27}$$

It is a linear function of Ω for $\hbar\Omega \ll k_B T$ and saturates at $-\tau/(\tau+\tau_s)\times\boldsymbol{\Omega}/\Omega$ at $\hbar\Omega \gg k_B T$ and $\tau_s \ll \tau$.[1]

The result above resembles the occupancy expected from a Boltzmann distribution, which may appear counterintuitive as the experimental splitting between the underlying states exceeds $k_B T$. However, the choice between Bose-Einstein and Boltzmann statistics is set by occupancies, not by the splitting. The Bose-Einstein distribution

$$f_{\mathrm{BE}}(\varepsilon) = \frac{1}{\exp\left(\frac{\varepsilon-\mu}{k_{\mathrm{B}}T}\right)-1}$$

reduces to the Boltzmann form

$$f_{\mathrm{B}}(\varepsilon) = \exp\left(\frac{\mu-\varepsilon}{k_{\mathrm{B}}T}\right)$$

when $\exp\left(-\frac{\mu-\varepsilon}{k_{\mathrm{B}}T}\right) \gg 1$, i.e., $f_{\mathrm{BE}}(\varepsilon) \ll 1$. Equivalently, the exciton density must satisfy

$$n \ll \frac{\hbar^2}{2m_0 k_{\mathrm{B}}T} \sim 10^{12}\ \mathrm{cm}^{-2},$$

with m_0 the exciton effective mass. The fluence used in the experiments results in $n \sim 10^9\ \mathrm{cm}^{-2}$, which is 3 orders of magnitude smaller, hence the Boltzmann approximation is appropriate.

To summarize the ensemble averaged longitudinal component of the pseudospin takes the form, see Eqs. (S9a) and (S27):

$$\boldsymbol{S}_{\parallel} = \frac{\tau}{\tau+T_{\parallel}}\left[T_{\parallel}\boldsymbol{G}_{\parallel} - \tanh\left(\frac{\hbar\Omega}{2k_B T}\right)\frac{\boldsymbol{\Omega}}{\Omega}\right]. \tag{S28}$$

where for convenience of comparison with experiments we introduced $T_{\parallel} = \tau_s$.

It is noteworthy that for thermalized ensemble of excitons, the ensemble averaging of Eqs. (S9) should be carried out. To avoid additional complications we assume that the pseudospin relaxation rates $\Gamma_{\alpha\beta}$ are appropriately averaged in Eqs. (S9).

[1]Note that according to Eq. (S6a) $\Gamma_{xx} = \tau_s^{-1}$ depends on Ω. Formally, for very large $\Omega\tau$ the relaxation time τ_s can exceed τ.

S1.3 Photoluminescence spectra of excitons in pseudomagnetic field

To derive compact analytical expressions for the emission spectra we develop a simplified model description of optically active exciton in 2D TMDC based on semiclassical (oscillator) model. Let $\mathbf{p}(t) = [p_x(t), p_y(t)]$ be the dipole moment of the exciton with $\boldsymbol{k} = 0$, i.e., the exciton which directly emits light. The components $p_\alpha(t)$ ($\alpha = x$ or y) obey the driven oscillator equations

$$i\dot{p}_\alpha = \omega_\alpha p_\alpha - i\gamma p_\alpha + \xi_\alpha. \tag{S29}$$

Here dot denotes the time derivative, ω_α is the exciton resonance frequency, $\omega_x \neq \omega_y$ is due to the strain-induced anisotropic splitting, γ is the exciton damping (for simplicity we disregard its dependence on α) and $\xi_\alpha(t)$ are the random sources related to the exciton relaxation from the high momentum to the optically active states. These random sources are the analogues of Langevin forces in the theory of fluctuations [10, 11]. The random sources approach can be justified for calculation of exciton emission spectra in the case of non-resonant pumping and linear-in-the-pumping regime [12, 13].

Let us calculate the emission spectrum using Eq. (S29). Since the Maxwell equations are linear, the spectrum of emitted electric field is proportional to the spectrum of $\mathbf{p}(t)$. We present

$$p_{\alpha,\omega} = \int_{-\infty}^{\infty} dt e^{i\omega t} p_\alpha(t) = \int_{-\infty}^{\infty} dt \int_{-\infty}^{\infty} dt_1 e^{i\omega t} G_\alpha(t - t_1)\xi_\alpha(t_1), \tag{S30}$$

where $G_\alpha(t)$ is the Green's function of Eq. (S29). The following correlator reads

$$\langle p_{\alpha,\omega} p^*_{\beta,\omega'} \rangle = \int_{-\infty}^{\infty} dt \int_{-\infty}^{\infty} dt_1 \int_{-\infty}^{\infty} dt' \int_{-\infty}^{\infty} dt_2 e^{i\omega t - i\omega' t'} G_\alpha(t - t_1) G^*_\beta(t' - t_2) \langle \xi_\alpha(t_1)\xi^*_\beta(t_2)\rangle, \tag{S31}$$

where the averaging takes place over the realization of the random sources. In the random sources approach, the correlations of the sources are given by the correlation matrix

$$\langle \xi_\alpha(t_1)\xi^*_\beta(t_2)\rangle = \delta(t_1 - t_2)\Xi_{\alpha\beta}, \tag{S32}$$

while other correlators, e.g., $\langle \xi_\alpha(t_1)\xi_\beta(t_2)\rangle$ are zero. The matrix elements of $\Xi_{\alpha\beta}$ can be related to the exciton generation rates in the density matrix approach, see below. Performing integrations, we find that

$$\langle p_{\alpha,\omega} p^*_{\beta,\omega'} \rangle = 2\pi\delta(\omega - \omega')(p_\alpha p^*_\beta)_\omega, \tag{S33}$$

where the spectrum is

$$(p_\alpha p^*_\beta)_\omega = \int_{-\infty}^{\infty} d\tau \int_{-\infty}^{\infty} dt e^{i\omega\tau} G_\alpha(\tau - t) G^*_\beta(-t)\Xi_{\alpha\beta} = G_{\alpha,\omega} G^*_{\beta,\omega}\Xi_{\alpha\beta}, \tag{S34}$$

with

$$G_{\alpha,\omega} = \frac{1}{\omega - \omega_\alpha + i\gamma}. \tag{S35}$$

Note that if the dynamics of dipoles is described by more complex linear equations with the matrix Hamiltonian H as $i\hbar\dot{p} = Hp$, the expressions (S34) are generalized, via the matrix Green's function G_ω, as

$$(p_\alpha p^*_\beta)_\omega = \sum_{\alpha'\beta'} G_{\alpha\alpha',\omega} G^*_{\beta\beta',\omega}\Xi_{\alpha'\beta'}, \quad G_{\alpha\beta} = [G]_{\alpha\beta}, \quad G = \frac{\hbar}{\omega I - H}, \tag{S36}$$

where I is the unit 2×2 matrix.

Now we turn to the calculation of the emission spectra. If we consider the analyzer at the angle φ, the emission spectra (up to a common factor) can be expressed as

$$I(\varphi) \propto \left(|E_x \cos\varphi + E_y \sin\varphi|^2\right)_\omega \propto \cos^2\varphi \left(|p_x^2|\right)_\omega + \sin^2\varphi \left(|p_y^2|\right)_\omega + \frac{\sin 2\varphi}{2}\left(p_x p_y^* + p_y p_x^*\right)_\omega . \tag{S37}$$

As a result, by virtue of Eq. (S34) the emission spectra measured in x, y and arbitrary polarizations can be expressed via the correlators of the random sources $\Xi_{\alpha\beta}$ as

$$I_x(\omega) = (|p_x|^2)_\omega = \frac{\Xi_{xx}}{(\omega-\omega_x)^2+\gamma^2}, \tag{S38}$$

$$I_y(\omega) = (|p_y|^2)_\omega = \frac{\Xi_{yy}}{(\omega-\omega_y)^2+\gamma^2}, \tag{S39}$$

$$\begin{aligned} I(\varphi) = {} & \cos^2\varphi \frac{\Xi_{xx}}{(\omega-\omega_x)^2+\gamma^2} + \sin^2\varphi \frac{\Xi_{yy}}{(\omega-\omega_y)^2+\gamma^2} \\ & + \sin 2\varphi \frac{\Xi_{xy}[\gamma^2 + (\omega-\omega_x)(\omega-\omega_y)]}{[(\omega-\omega_x)^2+\gamma^2][(\omega-\omega_y)^2+\gamma^2]}. \end{aligned} \tag{S40}$$

Here, for simplicity, we assumed that $\Xi_{xy} = \Xi_{yx}$. As we show below, it corresponds to the linearly polarized generation of excitons.

Let us now provide a relation between the random sources correlation matrix $\Xi_{\alpha\beta}$ and exciton generation rates in the density matrix approach which we used previously. To that end, we note that the single time correlators (mean squares, i.e., the Stokes parameters) can be expressed as[2]

$$\langle p_\alpha(t) p_\beta^*(t)\rangle = \int_{-\infty}^{\infty} \frac{(p_\alpha p_\beta^*)_\omega}{2\pi} d\omega. \tag{S41}$$

We obtain

$$\langle |p_\alpha(t)|^2\rangle = \frac{\Xi_{\alpha\alpha}}{2\gamma}, \tag{S42}$$

$$\langle p_\alpha(t) p_\beta^*(t)\rangle = \frac{\Xi_{\alpha\beta}}{2\gamma + i(\omega_\alpha - \omega_\beta)}. \tag{S43}$$

On the other hand, within the spin density matrix approach the average exciton occupancy

$$N = g\tau = \frac{1}{2}\left(\langle |p_x(t)|^2\rangle + \langle |p_y(t)|^2\rangle\right), \tag{S44}$$

where $\tau_{k=0} = 2\gamma$ is the exciton lifetime at $\boldsymbol{k} = 0$ state. Hence, the exciton generation rate g

$$g = \frac{\Xi_{xx} + \Xi_{yy}}{2}. \tag{S45}$$

[2]To that end it is sufficient to compare the definition: $\langle p_\alpha(t) p_\beta^*(t)\rangle = \int dt' G_\alpha(t-t') G_\beta^*(t-t') \Xi_{\alpha\beta}$ with the integral over ω of Eq. (S34).

At the same time, the pseudospin S components

$$S_x = \left(\langle |p_x(t)|^2\rangle - \langle |p_y(t)|^2\rangle\right), \tag{S46a}$$

$$S_y = \Big(\langle p_x(t)p_y^*(t)\rangle + \langle p_y(t)p_x^*(t)\rangle\Big), \tag{S46b}$$

$$S_z = \Big[\langle p_x(t)p_y^*(t)\rangle - \langle p_y(t)p_x^*(t)\rangle\Big], \tag{S46c}$$

obey the steady-state kinetic equation

$$\mathbf{S}\times\mathbf{\Omega} + \frac{\mathbf{S}}{\tau_{k=0}} = \mathbf{g}, \tag{S47}$$

where the vector generation rate $\mathbf{g}$ accounts for both induced and thermal polarization, cf. Notes S1.1 and S1.2.

Comparing the results of the solution of Eq. (S47) with Eqs. (S43), we obtain under linearly polarized excitation ($g_z \equiv 0$ and $\Xi_{xy} = \Xi_{yx}$)

$$S_x = \frac{1}{2}\tau_{k=0}(\Xi_{xx} - \Xi_{yy}) = \tau_{k=0}g_x, \quad S_y = \frac{1}{2}\Xi_{xy}\left(\frac{1}{2\gamma + i\Omega} + \frac{1}{2\gamma - i\Omega}\right) = \frac{g_y\tau_{k=0}}{1 + (\Omega\tau_{k=0})^2}, \tag{S48}$$

hence,

$$g_x = \frac{\Xi_{xx} - \Xi_{yy}}{2}, \quad g_y = \frac{\Xi_{xy} + \Xi_{yx}}{2}, \quad g_z = \frac{\mathrm{i}(\Xi_{xy} - \Xi_{yx})}{2}. \tag{S49}$$

Finally, we express the emission spectra Eq. (S38) in terms of generation rate Eqs. (S45), (S49) as

$$I_x(\omega) \propto (|p_x|^2)_\omega = \frac{g + g_x}{(\omega - \omega_x)^2 + \gamma^2}, \quad I_y(\omega) \propto (|p_y|^2)_\omega = \frac{g - g_x}{(\omega - \omega_y)^2 + \gamma^2}, \tag{S50}$$

or

$$I(\varphi) \propto N\cos^2\varphi \frac{g + g_x}{(\omega - \omega_x)^2 + \gamma^2} + N\sin^2\varphi \frac{g - g_x}{(\omega - \omega_y)^2 + \gamma^2} + N\sin 2\varphi \frac{g_y[\gamma^2 + (\omega - \omega_x)(\omega - \omega_y)]}{[(\omega - \omega_x)^2 + \gamma^2][(\omega - \omega_y)^2 + \gamma^2]}. \tag{S51}$$

We use the resulting dependence of the detected emission spectra on the analyzer angel φ to simulate the spectra of pseudo Zeeman split states in the Note S7.

S2 Calibration of strain

In this note we discuss our approach to define the strain tensor in the suspended monolayer. First, we note that the shift of exciton emission energy, ΔE, depends solely on the biaxial strain $(\varepsilon_{xx} + \varepsilon_{yy})/2$ and a material-specific constant A (Eq. (1) in the main text). This constant is determined through calibration measurements using a circular device, whose geometry simplifies the extraction of biaxial strain. With A known, we can estimate the biaxial strain in an arbitrary sample from the energy shift of its exciton emission line. Knowing the biaxial strain allows us to find the uniaxial strain, $\varepsilon_{xx} - \varepsilon_{yy}$, using the uniaxiality factor U defined in the main text. We quantitatively assess the strain in our devices using laser interferometry. The devices are modeled as optical cavities comprising a suspended two-dimensional material flake and the underlying Si/SiO_2 substrate (see Fig. S9a). The variation in the intensity of the reflected laser light as a function of membrane deflection is described by the following expression:

$$\Delta I_{laser} = \alpha(V_G) \sin\left(4\pi \cdot \frac{d(V_G)}{\lambda} + \varphi\right), \tag{S52}$$

where $d(V_G)$ represents the deflection of the suspended flake due to the applied gate voltage V_G, φ is the initial phase, and λ is the laser wavelength. The pre-factor $\alpha(V_G)$ accounts for the modifications in the optical properties of the 2D membrane under strain. Since the laser energy used in our experiments is approximately 0.6 eV away from the excitonic resonance of $MoSe_2$, strain-induced modulation of the band structure may cause minor changes in the optical constants. Thus, $\alpha(V_G)$ is approximated via expansion as $\alpha(V_G) = \alpha_0 + \alpha_1|V_G|$.

To relate the applied gate voltage V_G to the membrane deflection d, we consider the system's total energy U, which is the sum of the electrostatic and elastic energy components:

$$U = U_{\text{elec}} + U_{\text{elas}}.$$

Minimizing the total energy $\frac{\partial U}{\partial d} = 0$, following the approach in Ref. [14], yields the implicit relation between membrane deflection and gate voltage:

$$\frac{256 E_Y t \cdot d^3(V_G)}{9L^2} + \frac{16 E_Y t \varepsilon_0 \cdot d(V_G)}{3} - \frac{1}{2} C_G V_G^2 = 0. \tag{S53}$$

Here C_G is the capacitance of the system, E_Y is the Young's modulus of the membrane, t is its thickness, and ε_0 is the built-in (pre-)strain. The later can be found by comparing exciton emission energy in the regions of larger strain (center of the membrane) and regions with lower strain (supported on gold or edges of the membrane Fig. S1). By solving Eq. (S53), we obtain a fit function for the membrane deflection $d(V_G)$ as:

$$d(V_G) = \frac{\sqrt{h(V_G, a_1, a_2, \varepsilon_0)}}{3a_2 \cdot 2^{\frac{1}{3}}} - \frac{a_1 \cdot 2^{\frac{1}{3}}}{\sqrt{h(V_G, a_1, a_2, \varepsilon_0)}}, \tag{S54}$$

where a_1 and a_2 are material specific parameters, and the function $h(V_G, a_1, a_2, \varepsilon_0)$ is defined as:

$$h(V_G, a_1, a_2, \varepsilon_0) = \left(\sqrt{|108 a_1^3 a_2^3 + (27 a_2^2 |V_G|^2 - 27 a_2^2 \varepsilon_0)^2|} - 27 a_2^2 \varepsilon_0 + 27 a_2^2 |V_G|^2\right)^{\frac{1}{3}}.$$

Using Eq. (S52) and Eq.(S54), we fit the laser interference data (Fig. S9b) to extract the membrane deflection, shown by the blue line in Fig. S9c. We then relate membrane deflection to the biaxial strain in the suspended monolayer using a parabolic membrane approximation [14]:

$$\varepsilon = \frac{1+\varepsilon_0}{2}\sqrt{1+\frac{16d^2}{L^2}} + \frac{(1+\varepsilon_0)L}{8d}\log\left(\sqrt{1+\frac{16d^2}{L^2}}+\frac{4d}{L}\right) - 1, \tag{S55}$$

where L is the membrane diameter. The dependence of strain on gate voltage is presented in Fig. S9c, calculated using $L = 5.2\,\mu$m and $\varepsilon_0 = 0.2$. For small deflections, where strain is below 0.6%, the strain estimated from Eq. (S55) closely matches a simpler approximation $\varepsilon = 0.8\left(\frac{2d}{L}\right)^2$, with a discrepancy of less than 5% [15].

Next, using the extracted strain values, we determine the shift in the neutral exciton emission energy as a function of strain in $MoSe_2$ (Fig. S9d). The observed linear dependence of the emission energy on strain across the entire strain range confirms the accuracy of our strain measurements, as the conversion from the applied voltage to the induced strain is independent of the photoluminescence data. By fitting the data to a linear function, we find the parameter A of the Eq. (1) in the main text equivalent to the slope $A = \Delta E/\Delta\varepsilon = 36 \pm 4\,\text{meV}/\%$, which agrees with previous studies [16, 17].

Finally, we relate the extracted biaxial strain to the uniaxial strain present in the sample. For that we define the uniaxiality of the strain as $U = \frac{\varepsilon_{xx}-\varepsilon_{yy}}{\varepsilon_{xx}+\varepsilon_{yy}}$. This expression connects the biaxial and uniaxial strain as $\varepsilon_{xx} - \varepsilon_{yy} = U \cdot (\varepsilon_{xx} + \varepsilon_{yy})$. To determine the uniaxiality, we performed finite element analysis of the strain distribution in the sample (Fig. S1). The results show that the uniaxiality of the strain is around 80% and weakly depends on the biaxial strain remaining nearly constant across the applied strain range. We note that the COMSOL simulations were used only to estimate the uniaxiality of the strain, while the absolute values of the strain were determined directly using the interferometric measurements described above.

These simulated results can be approximated by simple analytical equation. We note that the strain along the major (a) and minor (b) axes of an ellipse is equivalent to the strain in circular membranes of the respective diameters. In a simple approximation, the strain in a circle is proportional to $\varepsilon \sim 1/D^2$, where D is the diameter. Therefore, the ratio of strain along the major and minor axis is $\varepsilon_{xx}/\varepsilon_{yy} = (a/b)^2$. For a typical device with $a = 8\,\mu$m and $b = 3\,\mu$m, the uniaxiality is close to the results of finite element simulation: $U = \frac{(a/b)^2-1}{(a/b)^2+1} \approx 80\%$.

Remarkably, the modeled uniaxiality depends weakly on the exact ratio of the major and minor axes; increasing U from 75% to 85% would require an approximately 25% increase in a to 10.5 μm with b fixed at $3\,\mu$m. We note that the exact shape of the membrane's edge as well as nonuniform distribution of the pre-strain may affect the uniaxiality of the strain.

Finally, we experimentally confirm that the uniaxial component of strain in our suspended membranes remains proportional to the total (biaxial) strain up to the largest values we apply. Following previous studies, we use polarization resolved Raman spectroscopy of the in plane E' phonon to track the strain anisotropy directly. Under uniaxial strain the doubly degenerate E' mode splits into two components whose atomic displacement vectors are parallel and orthogonal to the strain axis, whereas the out of plane A_1' mode remains essentially unaffected in energy. By analysing the polarization dependence of the Raman response, we separate these two E' branches and quantify their splitting, which provides an optical measure of uniaxiality.

Instead of $MoSe_2$ or WSe_2, where the E' peak is weak or spectrally convoluted with the A mode [18, 19], we perform these measurements on MoS_2, whose E' peak is strong and well isolated and for which extensive Raman literature exists [20, 21]. The optical setup mirrors that used for PL in the main text: a half-wave plate (HWP) placed in front of the objective co rotates the polarization of both the incident and the scattered light, allowing us to map the Raman response as a function of analyzer angle with respect to the strain axis.

Our observations are summarized in Fig. S10. At zero applied strain, the E' mode shows no resolvable splitting (Fig. S10a). Upon increasing strain, two clear effects appear (Fig. S10a): (i) both the E' and A modes shift in energy, as expected for tensile strain, and (ii) the E' mode energy becomes polarization dependent, while the A mode energy remains constant within our resolution. When we sweep the analyzer angle, the energy of the E' mode follows a characteristic four fold symmetry (Fig. S10b), exactly as predicted for a doubly degenerate in-plane vibration subjected to uniaxial strain [22]. This angular dependence enables us to assign the higher (lower) energy branch to vibrations parallel (orthogonal) to the strain axis, hereafter denoted E^+ and E^-.

To quantify how the uniaxial strain scales with the total strain, we record Raman spectra over a broad range of gate induced strain. The absolute value of the (biaxial) strain is obtained independently from interferometric calibration of membrane deflection (see Fig. S9). For each strain value we extract the energies of the E^+ and E^- peaks and plot their splitting versus the total strain (Fig. S5c). The energy of each mode changes linearly with total strain, demonstrating that the uniaxial component is linear with the overall deformation even deep in the high strain regime. Using literature values for the E′ splitting coefficient under pure uniaxial strain [21] and accounting for the strain distribution in our case, we convert the measured splitting into an uniaxial strain and then calculate the uniaxiality $U = (\varepsilon_{bb} - \varepsilon_{aa})/(\varepsilon_{bb} + \varepsilon_{aa})$ of our membrane. Above 0.5% total strain — where the two E' branches are well resolved — the extracted U is essentially constant and equal to ~ 0.75. This agrees with a simple geometric estimate for our elliptical trench (axes 3.3 and 8.3 μm; Fig. S10). This confirms that, across the entire strain range used in the main text, the ratio between uniaxial and biaxial components is determined by device geometry and does not change at high load.

S3 Analysis of Pseudospin Time Constants

In this note, we apply the pseudospin model developed in Note S1 to analyze the pseudospin distribution under uniaxial strain. Our objective is to relate the experimentally measured dependence of pseudospin on the pseudomagnetic field to the pseudospin relaxation time constants $T_{\|}$ and T_{coh}.

Longitudinal pseudospin component

We begin by relating the model of pseudospin component parallel to the pseudomagnetic field $S_{\|}$ Eq. (S28) to the experimental observations. The $S_{\|}$ is measured as the difference in emission intensities polarized parallel and perpendicular to the strain axis, defined as:

$$S_{\|} = \frac{I_{\|} - I_{\perp}}{I_{\|} + I_{\perp}}$$

here, $I_{\|}$ and $I_{\perp}$ are the intensities of neutral exciton emission with the analyzer set parallel and perpendicular to the strain axis x, respectively.

On a first glance the first term in Eq. (S28) is independent of the pseudomagnetic field, which implies a constant offset in $S_{\|}$ as a function of field. However, the density of photogenerated excitons in a specific polarization state — described by $G_{\|}$ — depends on the excitation wavelength. This is because the strain effectively increases the energy detuning between the excitation wavelength and the exciton energy, which reduces pseudospin polarization of excitons [23, 24, 25]. Therefore, we use high energy of laser (2.32 eV) to minimize the effect of G in the measurements of the $S_{\|}$. At low pseudomagnetic fields, $S_{\|}$ is less than 4%, primarily due to pre-strain effects (Fig. 3d of the main text). At higher fields, polarization retention becomes negligible, as confirmed by the independence of emission intensity on excitation polarization (Fig. S2c, Fig. S3e). Therefore, we further simplify Eq. (S28) by assuming $G_{\|} = 0$ and taking the absolute value of the pseudospin:

$$S_{\|} = \frac{\tau}{\tau + T_{\|}} \tanh\left(\frac{\hbar\Omega}{2k_B T}\right). \tag{S56}$$

Next, we consider the dependence of $T_{\|}$ on the pseudomagnetic field. According to Eq. (S8), the relaxation time along the field direction, $T_{\|} = 1/\Gamma_{xx}$, increases approximately quadratically with the field strength. Assuming that this time at zero pseudomagnetic field is equal to $\tau_{\mathrm{coh}} = 1/((\Omega_k^{\mathrm{LT}})^2 \tau_{\mathrm{sc}})$, measured in four-wave mixing experiments [26, 27], we derive the final equation:

$$S_{\|} = \frac{1}{1 + \frac{\tau_{coh}}{\tau}\left(1 + \frac{\Omega^2}{\tau_{coh}^2 (\Omega_k^{\mathrm{LT}})^4} + \sqrt{\left(1 + \frac{\Omega^2}{\tau_{coh}^2 (\Omega_k^{\mathrm{LT}})^4} + \frac{1}{\tau_{coh}^4 (\Omega_k^{\mathrm{LT}})^4}\right)^2 - \frac{4\Omega^2}{\tau_{coh}^4 (\Omega_k^{\mathrm{LT}})^6}}\right)} \tanh\left(\frac{\hbar\Omega}{2k_B T}\right). \tag{S57}$$

We fitted the experimental data by treating the temperature T and the root-mean-square exciton longitudinal–transverse splitting $\Omega^{\mathrm{LT}} = \sqrt{\langle \Omega_k^{\mathrm{LT}2} \rangle}$ (where angular brackets denote the averaging over the Boltzmann distribution of excitons) as fitting parameters, while adopting the exciton lifetime τ and coherence time τ_{coh} from the literature: for WSe_2, $\tau = 2$ ps and $\tau_{\mathrm{coh}} = 0.44$ ps; for $MoSe_2$, $\tau = 2$ ps and $\tau_{\mathrm{coh}} = 0.52$ ps [26, 27, 28, 29, 30, 31, 32]. The fit yielded values of $\Omega^{\mathrm{LT}} = 10.4 \pm 1.3$ T for WSe_2 and $\Omega^{\mathrm{LT}} = 12.0 \pm 1.1$ T for $MoSe_2$ in reasonable agreement with theoretical

predictions, see Note S4. The exciton temperatures were determined to be $T = 13.1 \pm 1.1$ K for WSe_2 and $T = 22.4 \pm 1.9$ K for $MoSe_2$, consistent with previous reports for optically initialized excitons [33, 34]. Given that the induced pseudomagnetic precession becomes significant above 5 T (where $\Omega\tau_{\text{coh}} > 1$), and that a constant $T_{\|}$ model yields unphysical values inconsistent with both theoretical constraints (see Eq. (S6)) and independent experimental results [35], we adopt the field-dependent model as the only physically consistent framework for describing the data.

Using Eq. (S7) and the extracted Ω^{LT}, we estimated the characteristic scattering time $\tau_{\text{sc}} = 1/(\tau_{\text{coh}}(\Omega_k^{\text{LT}})^2)$. We found $\tau_{\text{sc}} = 0.76 \pm 0.11$ ps for WSe_2 and 0.49 ± 0.05 ps for $MoSe_2$, values that are consistent with the theoretical estimates in Ref. [3], see also Note S4. Here we assumed that scattering time is constant for the entire range of pseudomagnetic field, unintended changes of Fermi energy may lead to slight variation of this time. A comparison between the simpler model with fixed $T_{\|}$ [Eq. (S56)] and the more complex model [Eq. (S57)] is shown in Fig. S11. These results constitute the first measurement of exciton longitudinal–transverse splitting in transition metal dichalcogenides. All the parameters are summarized in the table below.

	τ	τ_{coh}	Ω^{LT}	τ_{sc}	T
$MoSe_2$	2 ps	0.52 ps	12.0 ± 1.1 T	0.49 ± 0.05 ps	22.4 ± 1.9 K
WSe_2	2 ps	0.44 ps	10.4 ± 1.3 T	0.76 ± 0.11 ps	13.1 ± 1.1 K

We note that the lifetime of neutral exciton population could be longer than the values adopted from the literature, as typical measurements like time-resolved photoluminescence are not sensitive to hot excitons outside light cone [36]. Additionally, doping can modify the exciton lifetime by introducing new recombination pathways [30], and cavity effects—such as interference between the suspended monolayer and the substrate—can influence the radiative decay rate [37]. However, our extracted exciton longitudinal–transverse splitting Ω^{LT} is fairly insensitive to variations in the exciton lifetime. For instance, when we double the exciton lifetime τ of $MoSe_2$ to 4 ps, we find that Ω^{LT} decreases by approximately 10% to 10.9 T, which remains within the experimental error of our measurement.

Orthogonal pseudospin component

Next, we analyze the pseudospin component along the z-axis under pseudomagnetic field in the x direction when pseudospin along y-axis is initialized using linearly polarized light diagonal to the strain tensor axes. This component, S_z, describes the imbalance between σ^+ and σ^- emissions, and it is measured as

$$S_z = \frac{I_{\sigma^+} - I_{\sigma^-}}{I_{\sigma^+} + I_{\sigma^-}}.$$

We find that at low pseudomagnetic field S_z increases rapidly, and after ~ 4 T it starts to slowly decrease. In contrast, other excitons show lower S_z (Fig. S8). To compare this behaviour to theory use Eq. (S9b) with the result:

$$S_z = \frac{\tau_\perp^2 G\Omega}{1 + (\tau_\perp \Omega)^2}, \tag{S58}$$

where $\tau_\perp = 1/\sqrt{\tilde{\Gamma}_{yy}\tilde{\Gamma}_{zz}}$. This equation suggest that high initial polarization G of excitons is required to observe significant pseudospin rotation. Therefore, neutral exciton in WSe_2 is an

optimal system for observation of pseudospin effect due to large polarization retention even for excitation above the exciton resonance, unlike other excitonic states in this material or Mo-based materials [23, 24].

To quantitatively describe the dependence of S_z on the pseudomagnetic field, we first need to factor out the dependence of G on strain. To this end, we measure polarization-resolved photoluminescence (PL) of a circular sample, since in elliptical samples the pseudomagnetic field influences pseudospin dynamics (Fig. S3). Setting $\Omega = 0$ in Eqs. (S9), we find that the component of the pseudospin parallel to the field is $S^c = 2G\tau_\perp = G\tau_\parallel$, where $\tau_\parallel = 1/\tilde{\Gamma}_{xx}$, utilizing the relation $\Gamma_{zz} = 2\Gamma_{xx}$ at zero field [Eq. (S6)] and assuming that $\tau \gg \tau_\parallel, \tau_\perp$. We measure the linear polarization because the valley lifetime—which defines the retention of circular polarization (pseudospin along z) in WSe_2—is affected by dark excitons and resident carriers that can preserve the valley state for over 5 ps [35]; however, the coherence is lost on much shorter timescales ($<$ 0.5 ps) [23, 24]. To measure S^c, we excited the sample with linearly polarized light and measured the intensity of emission parallel and perpendicular to the excitation polarization. Hence,

$$G\tau_\perp = \frac{S^c}{2} = \frac{I_{\parallel,exc} - I_{\perp,exc}}{2(I_{\parallel,exc} + I_{\perp,exc})}, \tag{S59}$$

where $I_{\parallel,exc}$ and $I_{\perp,exc}$ are the intensity of the neutral exciton emission parallel and perpendicular to the polarization of the excitation. From the experimental data, we find that the polarization retention $G\tau_\perp$ decays exponentially with the strain (Fig. S6), which we attribute to the increasing separation between the laser wavelength and the exciton energy.

Finally, we find that ratio of S_z and S^c is described by the analog of Eq. (S58) free of generation term G:

$$S_z^* = \frac{\tau_\perp \Omega}{1 + (\tau_\perp \Omega)^2}. \tag{S60}$$

The equation suggests that the orthogonal pseudospin component S_z^* increases linearly at low pseudomagnetic fields, peaking at $\Omega\tau_\perp = 1$, and decreases for larger values of $\Omega\tau_\perp$. At higher fields, the pseudospin undergoes multiple rotations around the field $\mathbf{\Omega}$, resulting in a decreased of assymetry between σ^+ and σ^- emission. This behavior is consistent with the experimental data shown in Fig. 3 of the main text. Specifically, we obtained the data by dividing the pseudospin in the elliptical sample S_z (Fig. S6a) by the value measured in the circular device S^c (blue line in Fig. S6d). We then fitted the resulting S_z^* to Eq. (S60).

It is noteworthy that the dependence of the transverse relaxation time on the pseudomagnetic field is less pronounced than that of the longitudinal relaxation time $T_\parallel$. According to Eq. (S6), the relaxation rate along the z-axis is given by $\Gamma_{zz} = \Gamma_s \left(1 + \frac{1}{1+(\Omega\tau_{sc})^2}\right)$, where $\tau_{sc} = 1/(\tau_{\mathrm{coh}}(\Omega_k^{\mathrm{LT}})^2)$. Consequently, T_{coh} increases from $T_{coh} = \tau_{\mathrm{coh}}$ at low fields to $T_{coh} = 2\tau_{\mathrm{coh}}$ at high fields. Fitting the data with a field independent T_{coh} yields $T_{coh} = 0.8 \pm 0.2$ ps. In contrast, a more complex fit incorporates the effect of the depolarization field caused by the longitudinal-transverse splitting Ω_k^{LT}, Eq. (S8):

$$S_z^* = \frac{\tau^*\Omega}{1 + (\tau^*\Omega)^2}, \quad \tau^* = \frac{\tau\tau_{coh} f}{\tau + \tau_{coh} f}, \quad f = 1 + \frac{\Omega^2/\tau_{coh}^2(\Omega_k^{\mathrm{LT}})^4}{1 + \sqrt{\left(1 + \frac{\Omega^2}{\tau_{coh}^2(\Omega_k^{\mathrm{LT}})^4} + \frac{1}{\tau_{coh}^4(\Omega_k^{\mathrm{LT}})^4}\right)^2 - \frac{4\Omega^2}{\tau_{coh}^4(\Omega_k^{\mathrm{LT}})^6}}} \tag{S61}$$

Here, the value of Ω_k^{LT} is determined by the dependence of T_{coh} at high field, where the uncertainty is greater. Therefore, for the fitting procedure, we fixed Ω_k^{LT} to the value extracted from

the previous subsection, 10.4 T.

Figure S11 shows the comparison of the two models. The main difference between the two fits manifests as a slight divergence at high field strengths.

S4 Estimate of the exciton LT splitting

For a free monolayer the following expression for the neutral exciton LT splitting holds [38, 39, 40]

$$\Omega_k^{LT} = \frac{\Gamma_0}{q} k, \tag{S62}$$

where Γ_0 is the exciton radiative decay rate (at the in-plane wavevector $k = 0$, i.e., in the radiative cone), $q = \omega_0/c$ is the wavevector of photon emitted by the exciton, $\omega_0 = (E_g - E_B)/\hbar$ is the exciton transition frequency with E_g and E_B being the free-particle bandgap and exciton binding energy, respectively. Equation (S62) holds for $k \gg q$. In the presence of the screening induced, e.g., by the encapsulation and substrate one has instead of Eq. (S62)

$$\Omega_k^{LT} = \frac{\Gamma_0}{\varepsilon q} k, \tag{S63}$$

where ε is the effective dielectric constant of the surrounding, see Ref. [41] for details.

We are interested in Ω_k^{LT} averaged over the thermal distribution of excitons (this quantity enters the spin decay rates for thermalized excitons):

$$\bar{\Omega}_k^{LT} = \left[\int_0^\infty \frac{dE_k}{k_B T} e^{-E_k/(k_B T)} \left(\Omega_k^{LT} \right)^2 \right]^{1/2} = \frac{\Gamma_0}{\varepsilon q} \sqrt{\frac{2 M k_B T}{\hbar^2}}, \tag{S64}$$

where M is the exciton translational motion mass and T is the temperature.

Taking $\tau_r = 1/(2\Gamma_0) = 1$ ps, $M = 0.75 m_0$ (m_0 is free-electron mass), $T = 20$ K, $\varepsilon = 1$, and $E_g - E_B = 2$ eV we have

$$\bar{\Omega}_k^{LT} \approx 9 \text{ ps}^{-1}, \quad \hbar\bar{\Omega}_k^{LT} \approx 6 \text{ meV}, \quad \frac{\hbar\bar{\Omega}_k^{LT}}{2\mu_B} \approx 52 \text{ T}.$$

This sizable field corresponds to completely clean monolayer. The discrepancy between the estimate and the experimentally measured value may arise due to underestimation of dielectric constant or radiative lifetime [37]. For instance, residual layers of polydimethylsiloxane (PDMS) related to our fabrication technique, can increase ε to around 2.5 [42], reducing the effective field to $\Omega_k^{LT} \approx 21$ T.

At acoustic phonon scattering

$$\frac{1}{\tau_{sc}^{ac}} = C \frac{k_B T}{\hbar}, \tag{S65}$$

with the material-dependent coefficient $C = 0.5 \ldots 1$ [43, 44, 45] we have at $T = 20$ K (and $C = 1$)

$$\bar{\Omega}_k^{LT} \tau_{sc}^{ac} \approx 3.5 \ (@\ \varepsilon = 1) \qquad \text{or} \qquad \approx 1.3 \ (@\ \varepsilon = 2.5).$$

Hence, the product $\bar{\Omega}_k^{LT} \tau_{sc}^{ac}$ is not small, typically. Note that $\hbar\bar{\Omega}_k^{LT}$ is comparable with the mean kinetic energy of excitons which generally complicates the spin dynamics [46, 47].

S5 Calculation of Fermi energy

In this supplementary note, we determine the Fermi energy in suspended devices using two complementary methods. The first method is based on the dependence of the Fermi polaron-polariton binding energy on the applied gate voltage, while the second method uses the standard capacitor model to cross-validate the results.

The relationship between the binding energy of a Fermi polaron and the carrier density is well-established in the literature [48, 49, 50]. Briefly, the Fermi polaron is a quasiparticle formed by an exciton interacting with a Fermi sea of carriers, where the density of carriers governs the binding energy. This dependence is described by the equation:

$$E_{FP} = E_{FP}^0 + kE_F,$$

where E_{FP}^0 is the binding energy of the Fermi polaron at zero carrier density, and k is a constant. In our study, we use values that were measured for hBN encapsulated monolayers, $k = 1.1$, with $E_{FP}^0 = 26.1$ meV [49, 50]. The Fermi energy E_F, extracted as a function of applied gate voltage V_G, exhibits a near-linear dependence, as shown in Fig. S3f, consistent with theoretical expectations. Note that the binding energy of the FP in suspended samples E_{FP}^0 may differ from reported values due to differences in the dielectric environment for the suspended sample.

The carrier density n_e is related to the Fermi energy by the equation:

$$n_e = \frac{E_F m_e}{\pi \hbar^2},$$

where we assumed that the two valleys are occupied, and $m_e \approx 0.5 m_0$ is the electron effective mass, with m_0 being the free electron rest mass. For practical calculations, the relationship simplifies to:

$$n_e \approx 0.2 \times 10^{12} E_F, \tag{S66}$$

where units for n_e is cm^{-2} and E_F is meV.

This method assumes that the exciton-FP separation depends solely on doping; however, strain induced by the applied gate voltage may also affect the separation. Thus, a second method is required to validate the results.

We verify the results using a capacitor model to estimate the carrier density induced by the gate voltage. The carrier density $n_{e,h}$ in the membrane is approximated using a parallel plate capacitor model:

$$n_{e,h} = \frac{V_G \epsilon_0}{e} \left(\frac{\epsilon_{\mathrm{SiO_2}}}{d_{\mathrm{SiO_2}} + \epsilon_{\mathrm{SiO_2}} [d_{\mathrm{Au}} - d(V_G)]} \right),$$

where ϵ_0 is the vacuum permittivity, $\epsilon_{\mathrm{SiO_2}} = 3.6$ is the dielectric constant of SiO_2, e is elementary charge, $d_{\mathrm{SiO_2}} = 1000$ nm is the SiO_2 thickness, $d_{\mathrm{Au}} = 500$ nm is the distance between the gold surface and SiO_2, and $d(V_G)$ is the deflection of the membrane, determined via interferometry, see Note. S2. This model accounts for the increasing capacitance due to the deflection of the suspended flake. The induced carrier density can then be converted to Fermi energy using Eq. (S66).

Both methods show strong agreement (Fig. S3f), validating the accuracy of the exciton-FP splitting approach for determining the Fermi energy.

S6 Evaluation of Fermi polaron pseudo-Zeeman splitting

The theoretical curves presented in Fig. 4d of the main text are derived following Eqs. (31) and (33) of Ref. [51]. For $MoSe_2$, the ratio between the neutral exciton and Fermi polaron pseudo-Zeeman splittings is given by

$$\frac{\hbar\Omega_{FP}}{\hbar\Omega_X} = \frac{\left(M_X/M_T\right)^3}{4\,\sinh^2\left[\frac{1}{2}(M_X/M_T)^2\right]}\frac{E_F}{E_b}, \tag{S67}$$

where M_X and $M_T = \frac{3}{2}M_X$ denote the exciton and Fermi polaron masses, respectively, and $E_b = 26.1\,\mathrm{meV}$ is the binding energy relative to the exciton. Note that the equation is valid only at $E_F \ll E_b$.

In WSe_2, the Fermi polaron splitting is described by

$$\frac{\hbar\Omega_{FP}}{\hbar\Omega_X} = \frac{\left(M_X/M_T\right)^3}{2\,\sinh^2\left[\frac{1}{2}(M_X/M_T)^2\right]}\frac{E_F}{\Delta}, \tag{S68}$$

where $\Delta = 6.8\,\mathrm{meV}$ is the energy difference between intra- and inter-valley trion states [50]. Since $\Delta \ll E_b$ the FP splitting in WSe_2 is larger for the same field strength and Fermi energy.

S7 Simulation of pseudo-Zeeman splitting at large pseudomagnetic fields

In this section, we provide details on the simulation of pseudo-Zeeman splitting used in the main text and compare the resulting behavior with experimental data. We show that thermal depletion of the higher-energy pseudo-Zeeman split state sufficiently explains the saturating dependence of the exciton splitting at high pseudomagnetic fields (Fig. 2c of the manuscript). We briefly summarize the experimental procedure and then describe the simulation approach that we used to best match the experiment.

In short our experiential procedure is the following. We use linearly polarized light to excite a pseudospin in a specific state on the Bloch sphere (see Fig. 1c of the main text). This pseudospin undergoes damped Larmor precession toward the pseudomagnetic field direction and eventually radiatively decays, imprinting its state onto the photoluminescence spectra. We then employ a linear polarizer (analyzer) to isolate the emission of a specific pseudospin state on the Bloch sphere. The pseudo-Zeeman splitting manifests as a difference in the emission energy of the two pseudospin states aligned along and opposite to the pseudomagnetic field.

To map this splitting, we measure the emission spectra of the sample as a function of the analyzer angle, keeping the excitation and detection polarizations parallel to reduce the influence of the pseudospin generation rate ($\boldsymbol{G}$ in Eq. (S9)). Each spectrum is fitted with Gaussian functions to extract peak positions at each angle, and the resulting dependence is analyzed using $E = E_0 + \hbar\Omega/2\cos\varphi$ to extract the pseudo-Zeeman splitting.

In our simulations, we aim to closely replicate the experimental procedures. First, we calculate the amount of pseudospin generated in the $MoSe_2$ sample using: $N = \frac{P\sigma\lambda 2\pi\tau}{\hbar c\pi r^2}$, where $P = 1\,\mu\text{W}$ is the laser power, $\sigma = 5\%$ is the absorption coefficient, $\lambda = 680\,\text{nm}$ is the excitation wavelength, $r = 0.5\,\mu\text{m}$ is the laser spot radius, and $\tau = 2\,\text{ps}$ is the exciton lifetime. The generation rate of pseudospin lies in the xy-plane, forming an angle 2φ with the pseudomagnetic field direction, and is set to 3% (see Fig. S3) of the total exciton density, $G = 0.03N/\tau$.

We then calculate the steady-state pseudospin components using Eqs. (S9) with experiment informed parameter: $\tau = 1.8\,\text{ps}$, $T_{\parallel} = 10\,\text{ps}$, $T_{coh} = 0.8\,\text{ps}$, and $T = 10\,\text{K}$. Here we used the experimentally obtained parameters for the model with relaxation times independent of pseudomagnetic field [Eq. (S56), (S60)] to simplify the model. Subsequently, we compute the spectra at each analyzer angle φ using Eq. (S51), accounting for an approximate 7% loss of polarization observed in these experimental settings. To model the unpolarized background likely arising from a tail of charged excitons (see spectra below 1.6 eV in Fig. S12a), we add a broad unpolarized Gaussian peak 22 meV below the exciton, with strain-dependent intensity reproducing the experimental behavior. The resulting data are then fitted with Gaussian functions to extract the peak positions, which are analyzed as a function of angle using the same equation as in the experiment.

The simulation results at a large pseudomagnetic field closely match the experimentally observed exciton splitting (Fig. S12a). Notably, while we used a pseudo-Zeeman splitting of 2.5 meV in the simulations, the exciton splitting extracted with method described above is approximately 0.8 meV. This discrepancy primarily arises from an underestimation of the emission energy of the higher-energy state due to thermal depletion (see Eq. (S28)). Although the extraction of splitting could potentially be improved by fitting two peaks for the split excitons, this approach would significantly reduce the stability of the procedure, given that the splitting is much less than the

width of the peaks.

We further compare the experimentally extracted splitting with the values obtained from simulations at different pseudomagnetic fields (points and solid line in Fig. S12b). The close agreement between the experiment and simulation supports the assumption of thermal depletion of the higher-energy state at high pseudomagnetic fields. Additionally, we compare the experimentally measured pseudospin polarization $S_{\parallel}$ with the simulation output (Fig. S12c). To satisfy experimental conditions, we conducted a separate set of simulations with a fixed direction of the generation term $\boldsymbol{G}$. The simulated splitting increases linearly at low fields and saturates at high pseudomagnetic fields, consistent with the experimental data.

We note that while the model accurately describes the behavior of the pseudo-Zeeman splitting, it is qualitative in nature, as it involves simplified background emission.

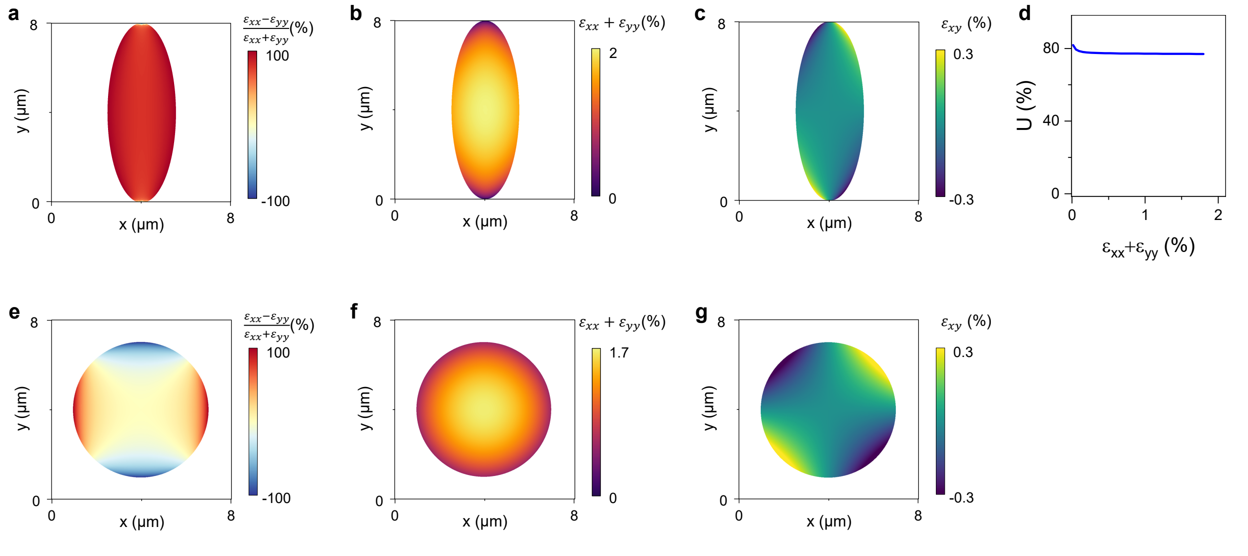

Figure S1: **Strain profile in elliptical and circular devices.** **a-c**: False color maps of strain uniaxiality $\frac{\varepsilon_{xx}-\varepsilon_{yy}}{\varepsilon_{xx}+\varepsilon_{yy}}$, total strain ($\varepsilon_{xx}+\varepsilon_{yy}$), and shear strain ($\varepsilon_{xy}$) in an elliptical device simulated using finite element analysis. Simulations were performed using COMSOL Multiphysics software, employing the electrostatics and structural mechanics modules. Using the electrostatic module we calculate the electrostatic pressure on the membrane, which was then applied in the structural mechanics module to simulate the membrane's response. The parameters used in the simultion of the TMD membrane were set to a thickness of 0.7 nm, Poisson's ratio of 0.19, density of 9.32 g/cm^3, and Young's modulus of 117 GPa [52, 53]. Pretension was applied as an in-plane initial stress of 15 MPa, resulting in a strain of $\varepsilon_0 \sim 0.1\%$, consistent with experimental values. The total strain $\varepsilon_{xx}+\varepsilon_{yy}$ is homogeneous at the center of the device, with variation less than $\frac{\Delta\varepsilon}{\varepsilon} < 10\%$ within a 1 μm diameter region. In principle, shear strain may contribute to a more complex pseudomagnetic field (see Note S1), simulations indicate it is negligible at the device center. Note that the color scale in graph (a) differs from that in Fig. 1f of the main text for better comparison with the circular device. **d**: Dependence of strain uniaxiality U on total strain at the center of the elliptical sample. Throughout the range of applied strain, the uniaxiality remains approximately 80%. **e-g**: False color maps of strain uniaxiality, total strain, and shear strain in a circular device. As expected from the device's symmetry, the uniaxiality of strain is zero at the device center.

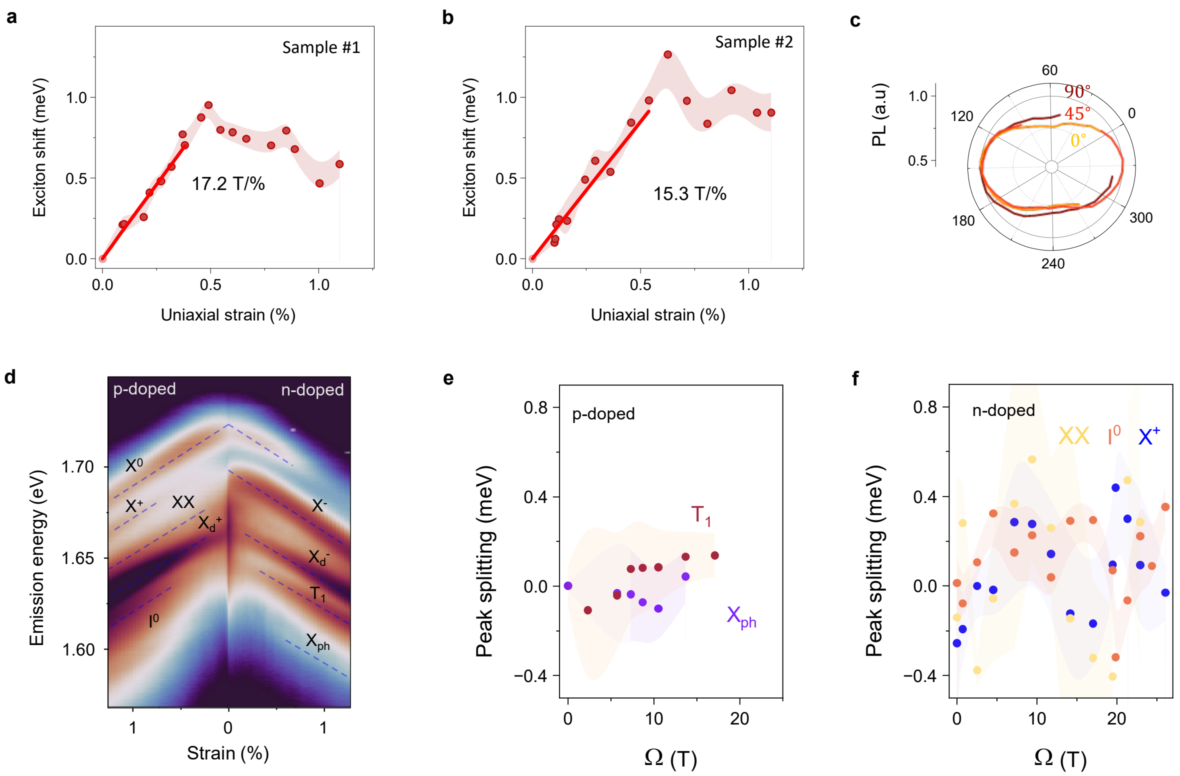

Figure S2: **Extended analysis of WSe_2 device.** **a,b**: Shift of the exciton emission energy in WSe_2 devices #1 (Fig. 3b,c of the main text) and #2 (Fig. 3d and Fig. 4 of the main text) as a function of applied uniaxial strain. The exciton shift is derived from the dependence of exciton emission energy on analyzer angle, following the procedure outlined in the main text. The strain-to-pseudomagnetic field conversion factor is extracted from a linear fit to the linear region of the data and determined to be 16.1 ± 1.8 T/%. Note that the largest uniaxial strain obtained in the WSe_2 devices is $\sim 1.5\%$, but intensity of neutral exciton emission is too low to extract the exciton splitting due to effect of doping. **c**: Dependence of the neutral exciton emission intensity in WSe_2 on analyzer angle for a 2.32 eV excitation polarized at $\sim 0°$, 45°, and 90° relative to the pseudomagnetic field (yellow, orange, and red lines, respectively). The pseudomagnetic field favors emission polarized along the $\sim$ 150° direction, coinciding with the minor axis of the ellipse (which is the direction of the $\mathbf{\Omega}$). The emission pattern shows minimal dependence on excitation polarization, indicating low G, as the laser energy is significantly detuned from the exciton energy. **d**: False-color PL map of the strained elliptical sample for both polarities of the gate voltage (V_G), plotted on a logarithmic scale. In the negative voltage region (p-doped), we observe the neutral exciton (X^0), positively charged exciton (X^+), biexciton (XX), dark charged exciton (X_d^+), and the I^0 peak, commonly attributed to phonon replica of the intervalley dark exciton. For positive voltage (n-doped), additional features such as the negatively charged bright and dark excitons (X^-, X_d^-), the T_1 peak (origin debated), and the phonon replica peak (X_{ph}) are identified. Due to broad linewidths, only one X^- peak is observed, likely a combination of singlet and triplet states, dominated by the brighter triplet. The strain-induced energy shift is symmetric with respect to V_G polarity. **e-f**: Peak splitting of excitons omitted in the main text in p-doped and n-doped regimes, respectively. In the p-doped regime, negligible splitting is observed for the X_{ph} peak, a finite splitting of the T_1 peak is detected, though it remains comparable to noise. In the n-doped regime, splitting of the XX, I^0, and X^+ peaks exhibit larger noise due to their low intensity relative to X_d^+, alongside significant peak broadening.

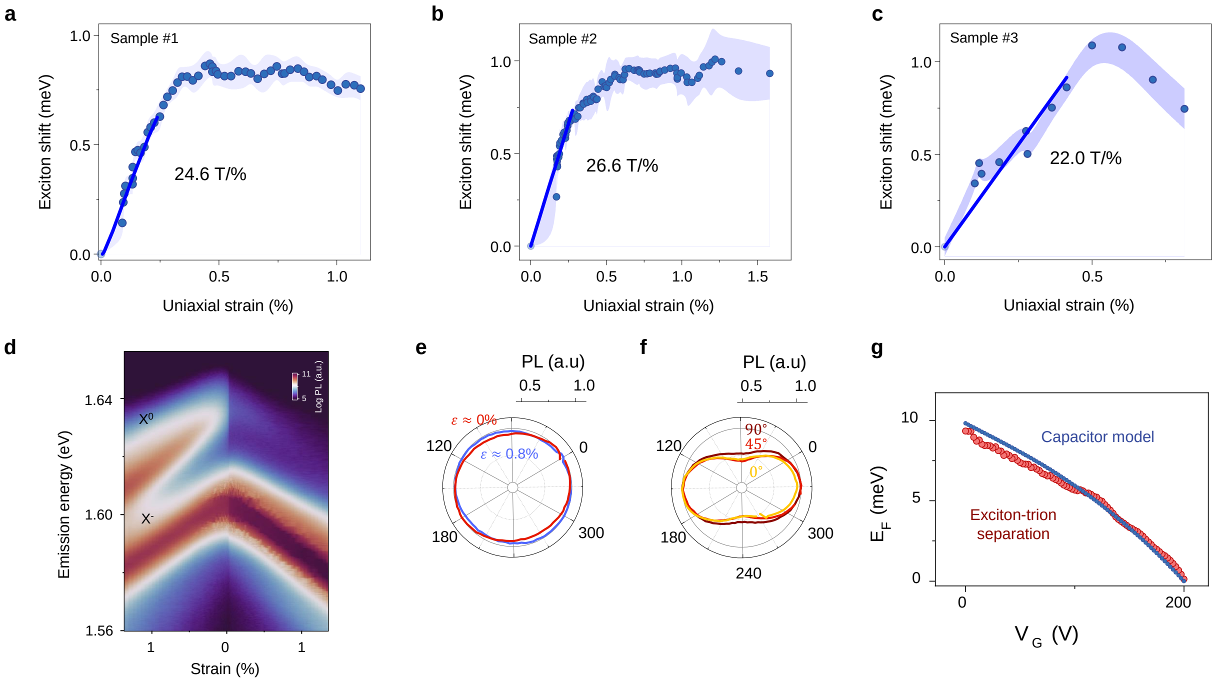

Figure S3: **Extended analysis of $MoSe_2$ device.** **a-c**: Shift of the exciton emission energy in $MoSe_2$ devices #1 (Fig. 2, 3, 4d of the main text), #2, and #3 as a function of applied uniaxial strain. The exciton shift is extracted from the dependence of exciton emission energy on analyzer angle, following the procedure outlined in the main text. The strain-to-pseudomagnetic field conversion factor is obtained from a linear fit to the linear region of the data. The average conversion factor is found to be 24.6 ± 2.5 T/%. **d**: False-color PL map of the strained $MoSe_2$ sample for both polarities of the gate voltage (V_G), plotted on a logarithmic scale. **e**: Dependence of neutral exciton emission intensity in a circular $MoSe_2$ membrane on analyzer angle at 0% applied strain (red) and at $\varepsilon_{xx} = \varepsilon_{yy} = 0.8\%$. A slight dependence of emission intensity on the analyzer angle is observed at zero strain, likely due to the retention of initial polarization. However, the lack of angular dependence at finite biaxial strain in circular device confirms that pseudospin polarization in elliptical sample is induced by the pseudomagnetic field. **f**: Dependence of neutral exciton emission intensity in an elliptical $MoSe_2$ membrane on analyzer angle for 1.83 eV excitation polarization at $\sim 0°$, $45°$, and $90°$ relative to the pseudomagnetic field (yellow, orange, and red lines, respectively). The pseudomagnetic field favors emission polarized along the $\sim 150°$ direction, which coincides with the minor axis of the ellipse. The emission pattern shows minimal dependence on the excitation polarization, indicating low retention of initial polarization, as the laser energy is well detuned from the exciton energy. **g**: Comparison of Fermi energy dependence on gate voltage (V_G) extracted using exciton-polaron energy separation (red dots) and a capacitor model (blue points). For exciton-polaron separation, the model developed in ref.[48, 49, 50] is employed (see Note S5). To cross-check the results, an independent method—based on a capacitor model—is used (Note S5). Both methods yield consistent results, confirming the validity of the approaches.

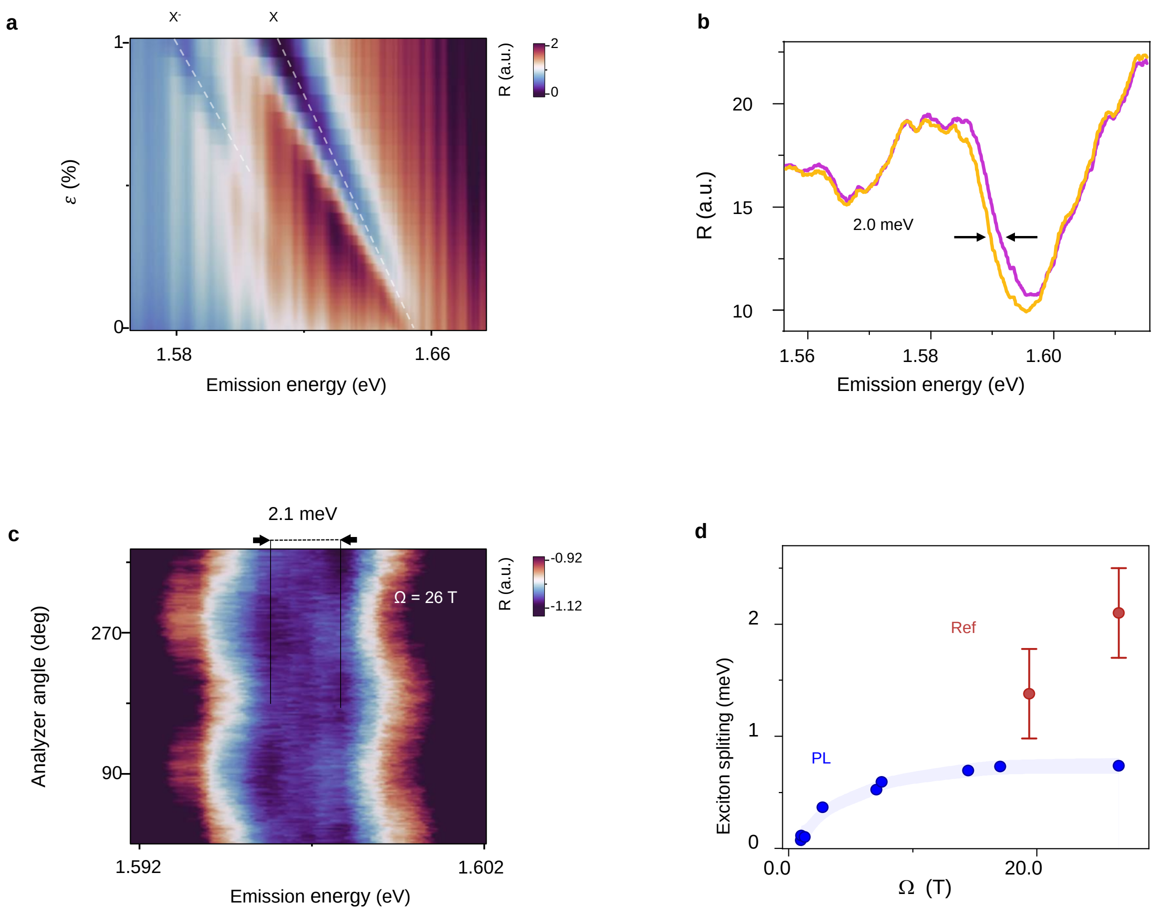

Figure S4: **Exciton Pseudo-Zeeman splitting measured in reflectivity a:** White-light reflectivity of a suspended monolayer $MoSe_2$ as a function of strain. The neutral- and charged-exciton resonance red-shifts by approximately 50 meV at 1.0% strain. We note that the interference effect in the cavity prevents us from measuring differential reflectivity. **b:** At 1.0% strain, polarization-resolved reflectivity reveals a clear splitting of the exciton peak. Traces recorded with detection along the ellipse's minor (purple) and major (orange) axes show a 2.0 meV separation larger than the value inferred from PL. **c:** Analyzer-angle-resolved reflectivity at the same strain confirms a pseudo-Zeeman splitting of around 2.1 meV as measured by energy separation beween the minimas of the reflectivity curves. **d:** Exciton splitting extracted from PL (blue) and reflectivity (red) versus pseudomagnetic field: reflectivity better captures the expected splitting. We attribute it to direct nature of this probe; unlike PL, it is not biased by the "dimming" of the higher-energy branch of the pseudo-Zeeman split state.

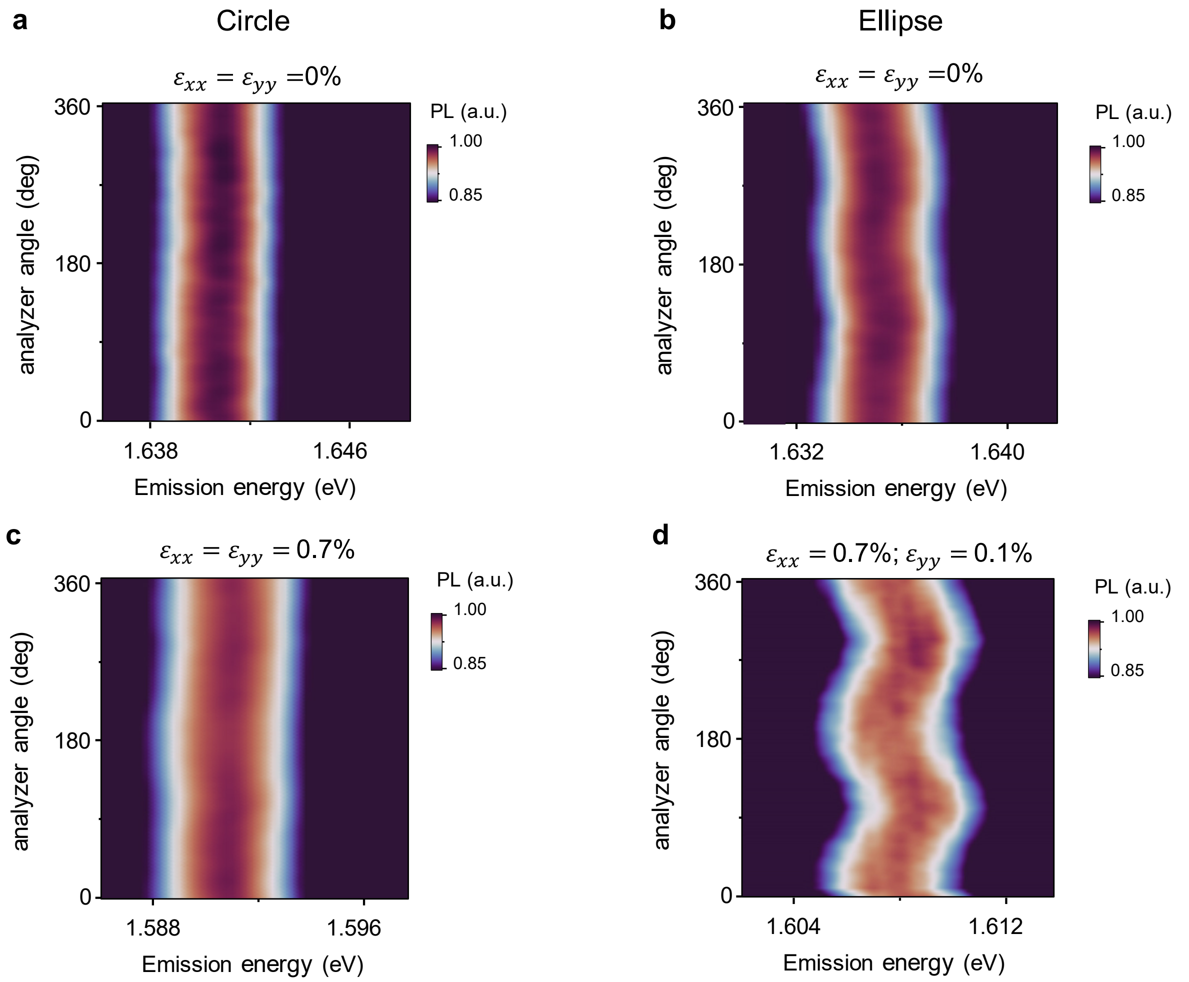

Figure S5: **Biaxial vs. uniaxial strained $MoSe_2$ a-b:** False-color map of neutral exciton PL in a circular and elliptical $MoSe_2$ membrane as a function of analyzer angle at 0% applied strain. Some dependence of emission intensity on the analyzer angle is assigned to prestrain that is estimated by comparison to the emission at the sample edge $\varepsilon_0 = 0.08\%$ in the elliptical sample. **c-d:** False-color map of neutral exciton PL in the same two devices under finite strain. This results in strain tensor $\varepsilon_{xx} = \varepsilon_{yy} = 0.7\%$ in circular membrane and $\varepsilon_{xx} = 0.7\%; \varepsilon_{yy} = 0.1\%$ in elliptical one. The emission intensity in the circular sample shows no dependence on the analyzer angle, while the elliptical sample exhibits a clear angular dependence, indicating the presence of a pseudomagnetic field. The pseudomagnetic field is oriented along the minor axis of the ellipse, as expected.

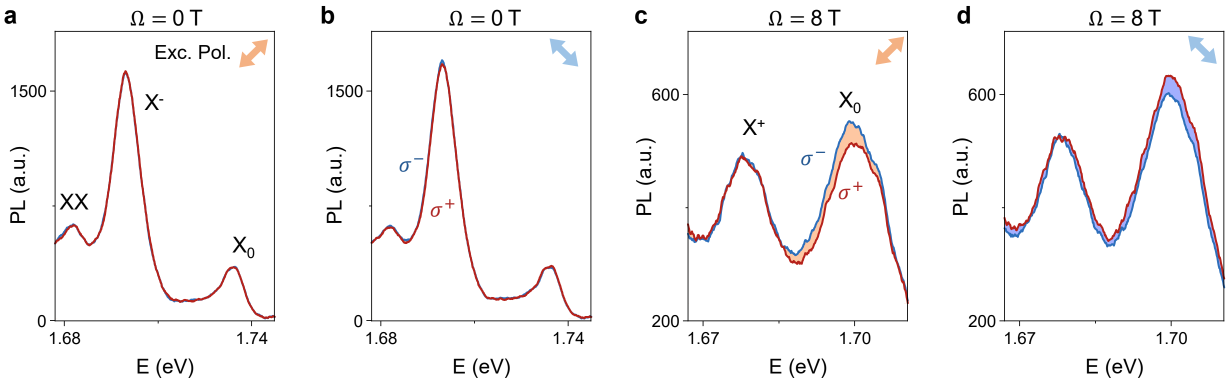

Figure S6: **Emergence of orthogonal pseudospin component in pseudomagnetic field a,b:**. Polarization resolved PL spectra of pristine WSe_2 monolayers under linearly polarized excitation at angles of +45° (a) and 45° (b) relative to the strain axis. The spectra exhibit negligible variation in emission intensity between σ^+ (red) and σ^- (blue) polarized light, suggesting absence of pseudospin rotation in the absence of external pseudomagnetic fields. **c,d:** Polarization resolved PL spectra under an applied pseudomagnetic field of $\Omega = 8$ T, showing significant asymmetry in σ^+ and σ^- polarized emission. Reversing the excitation polarization between +45° (c) and -45° (d) results in a sign flip of the asymmetry, consistent with pseudospin rotation induced by the pseudomagnetic field. Notably, slight differences in the spectra for the two excitation polarizations are observed; to mitigate setup-related effects, we average the extracted pseudospin values S_z across both excitation polarizations.

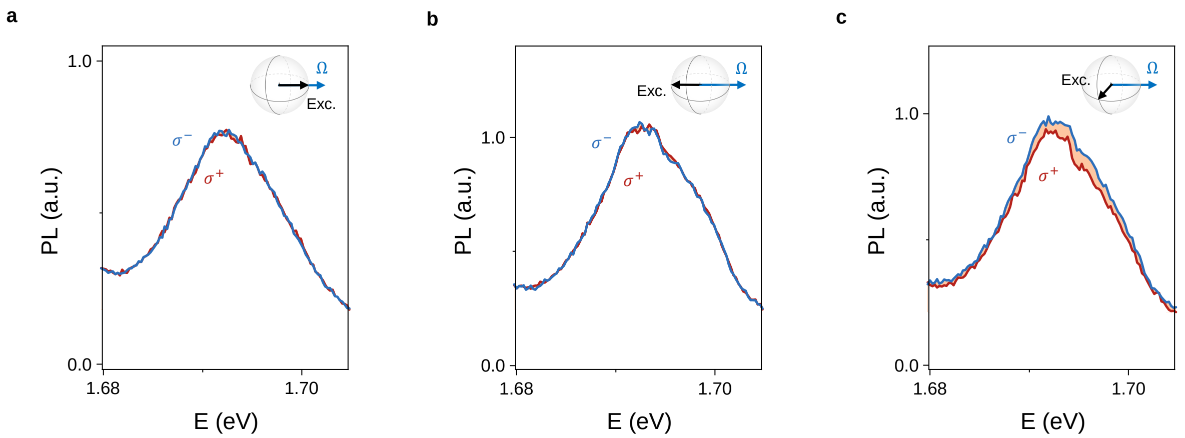

Figure S7: **Pseudo-Larmor effect in WSe_2 at various polarizations of excitation a-b:** PL spectra of WSe_2 (distinct from the sample in Fig. 3 of the main text) at $\Omega = 8$ T under linear excitation aligned (a) parallel and (b) antiparallel to the pseudomagnetic field show no circular polarization imbalance, as expected when the pseudospin is parallel to Ω. Insets depict excited pseudospin orientation relative to Ω in each case. **c:** When the sample is rotated to create a 45° angle between excitation and Ω, a σ^-/σ^+ asymmetry appears, consistent with pseudo-Larmor precession. The asymmetry is expected to be proportional to the projection of the pseudospin onto the Ω axis, which is maximal at 45°.

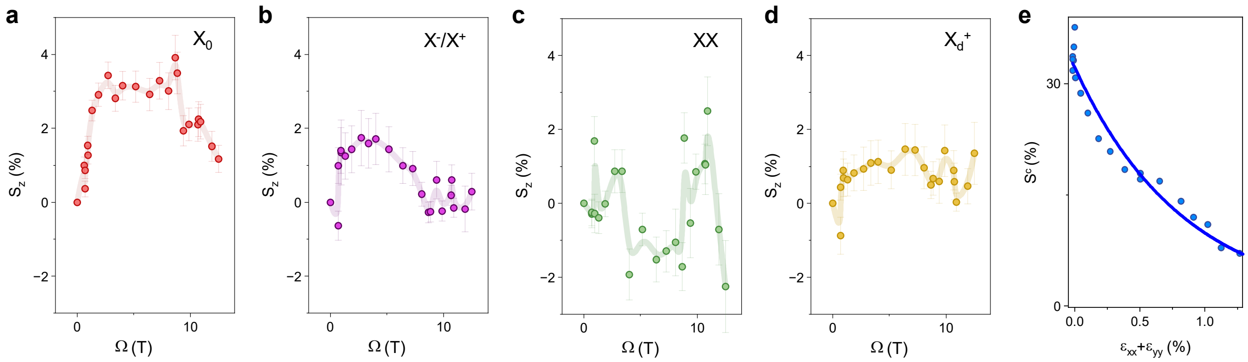

Figure S8: **Dependence of S_z of X_0, X^-, XX, X_d^+ on pseudomagnetic field a-d:** Orthogonal pseudospin component S_z of neutral excitons (X^0), bright charged excitons (X^-/X^+), biexciton (XX), and dark charged excitons (X_d^+) in WSe_2 as a function of the pseudomagnetic field. The solid splines serve as a guide to the eye. The largest signal is observed for neutral exciton, reflecting its higher generation G (see Eq. (S58)). We also observe non-zero S_z for charged excitons that peaks at lower pseudomagnetic field compared to neutral excitons. The study of it is complicated due to doping related change of G. Other species show a smaller signal close to the noise floor. We note that the device used in the measurement had slight initial doping, leading to a transition from negative trion to positive trion at around $\Omega \approx 2.5$ T. **e:** Pseudospin of neutral exciton $S^c = \frac{I_{\parallel,exc} - I_{\perp,exc}}{(I_{\parallel,exc} + I_{\perp,exc})}$ (Note S3) in a circular WSe_2 membrane (points). The reduction in pseudospin value is primarily due to the increasing separation between the laser wavelength and the exciton energy[25, 23], which we fit to an exponential decaying function (solid line). The measurement was conducted in a circular device to avoid effects of pseudomagnetic fields on pseudospin.

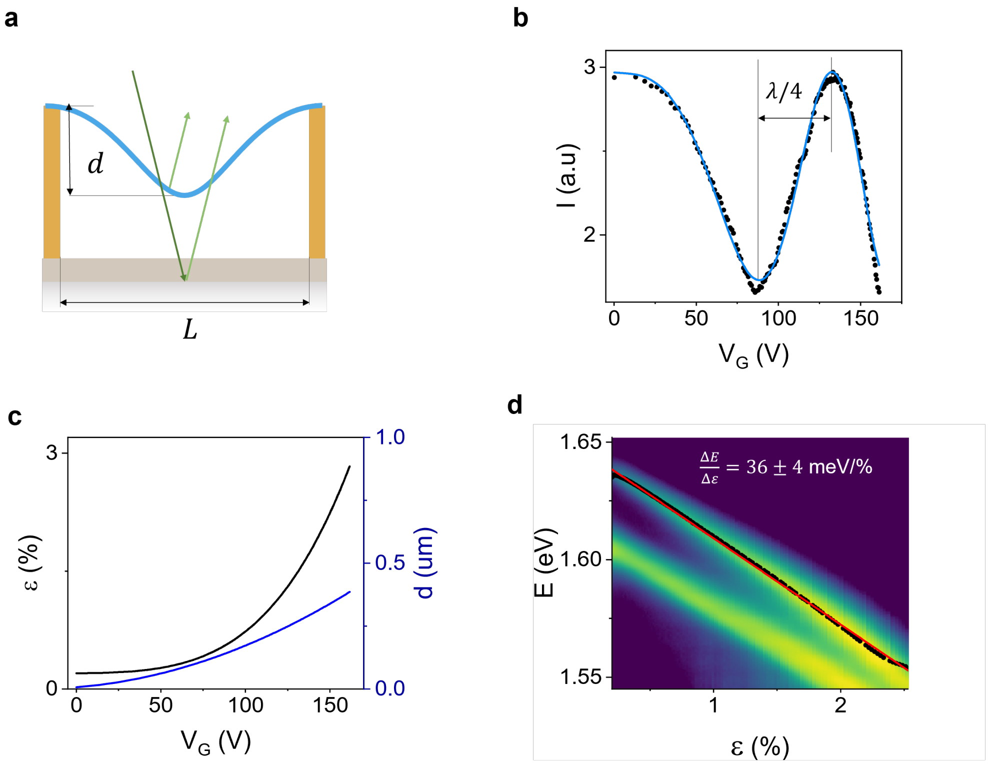

Figure S9: **Strain calibration a:** Schematic of the experimental setup used to measure strain in the device. The phase difference between the laser reflected off the membrane and the Si substrate is sensitive to membrane deflection, leading to optical interference. **b:** Deflection of the membrane as a function of gate voltage (dots) fitted to the theoretical model (blue line). The intensity modulation of reflected light corresponds to deflections equal to multiples of the laser wavelength. **c:** Deflection of the membrane (right axis, blue line) and strain $\varepsilon_{xx} + \varepsilon_{yy}$ (left axis, black line) as a function of gate voltage. **d:** False-color map of photoluminescence spectra of a $MoSe_2$ monolayer as a function of applied strain. The relationship between the excitonic energy shift and strain is determined by a linear fit (red line) to the shift of neutral exciton emission energy (black dots).

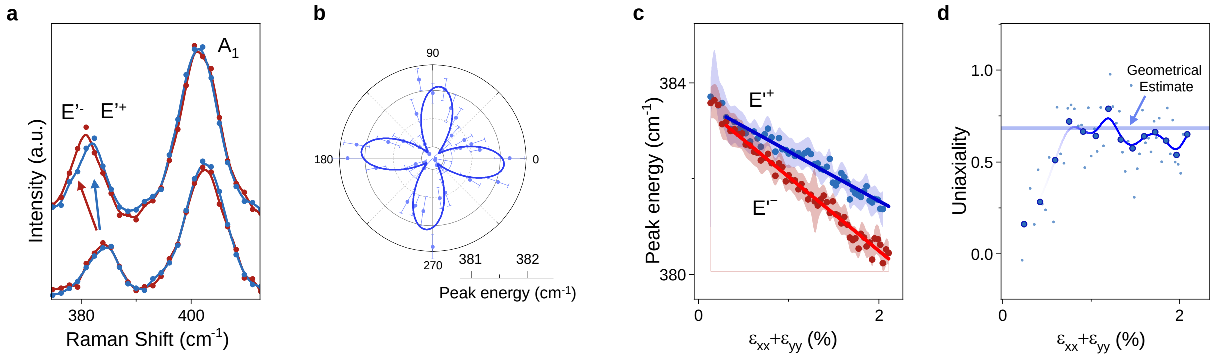

Figure S10: **Quantification of uniaxial strain using polarization-resolved Raman. a:** Representative Raman spectra of the in-plane E' and out-of-plane A_1 Raman modes under tensile uniaxial strain. The doubly degenerate E' mode splits into E^+ (parallel) and E^- (orthogonal) to the strain axis, while A_1 remains essentially unchanged. **b:** Angular dependence of the E' peak energy versus analyzer angle exhibits the expected four-fold symmetry for an uniaxially split E' mode. **c:** Peak energies of E^+ and E^- versus total (biaxial) strain show linear trends, supporting linearity of uniaxial strain in the entire measurement range. **d:** Uniaxiality U extracted from the E' splitting (small markers) is approximately constant above $\sim 0.5\%$ total strain, when peaks are well resolved. The large markers show a 5-point moving average and the curved line is a guide to the eye. The data agree with the simple geometric estimate $U = (b^2 - a^2)/(b^2 + a^2)$, where $a = 3.3\ \mu$m and $b = 8.3\ \mu$m are the axes of elliptical trench (horizontal line), confirming that the uniaxial-to-biaxial strain ratio is fixed by device geometry and does not degrade at high load.

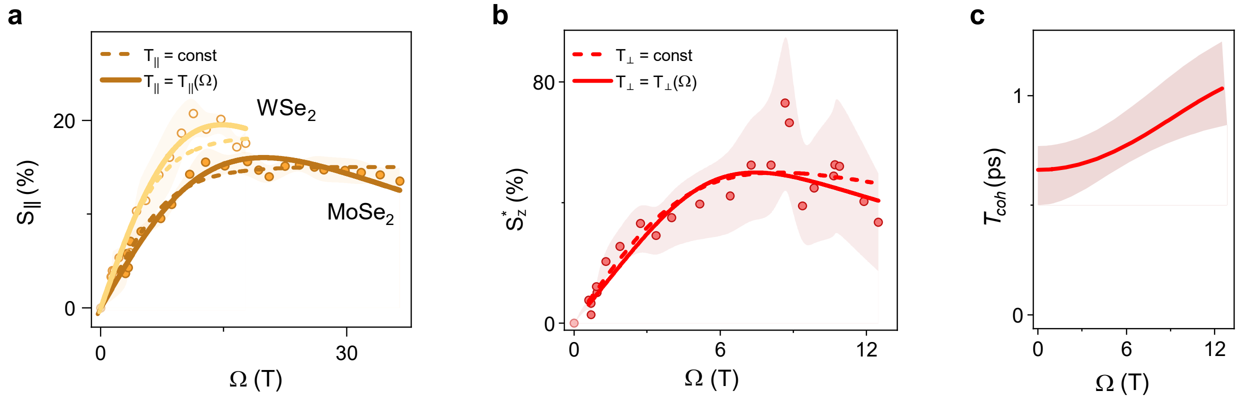

Figure S11: **The effect of pseudomagnetic field on relaxation times a:** The component $S_{\parallel}$ of the pseudospin as a function of the pseudomagnetic field in $MoSe_2$ and WSe_2 (filled and empty circles, respectively), along with fits to the simple model Eq. (S56) with fixed $T_{\parallel}$ (dashed lines) and the more complex model (S57) where $T_{\parallel}$ is a function of the field. The simple model yields $T_{\parallel} = 10.2$ ps for $MoSe_2$ and $T_{\parallel} = 8.9$ ps for WSe_2, using $\tau = 1.8$ ps and $\tau = 2$ ps, respectively, and $T \approx 5$ K. Note that while the simple model with fixed relaxation times fits the data well, it yields the times inconsistent with results of $T_{\perp}$, as it violates Eq. (S6): $T_{\parallel}(\Omega = 0) = T_{\perp}(\Omega = 0)/2$. **b:** The component S_z of the pseudospin as a function of the pseudomagnetic field strength in WSe_2, along with fits to the simple model Eq. (S60) with fixed T_{coh} (dashed lines) and the more complex model Eq. (S61) where T_{coh} is a function of the field. **c:** The dependence of the transverse relaxation time T_{coh} on pseudomagnetic field strength. The slightly higher value of $T_{coh} = 0.68$ ps at low field compared to results of four wave mixing experiments at zero field $\tau_{\mathrm{coh}} = 0.44$ ps [23, 24] could be related to preservation of valley degrees of freedom by dark excitons and resident carriers, effectively prolonging the lifetime of the pseudospin along the z-axis.

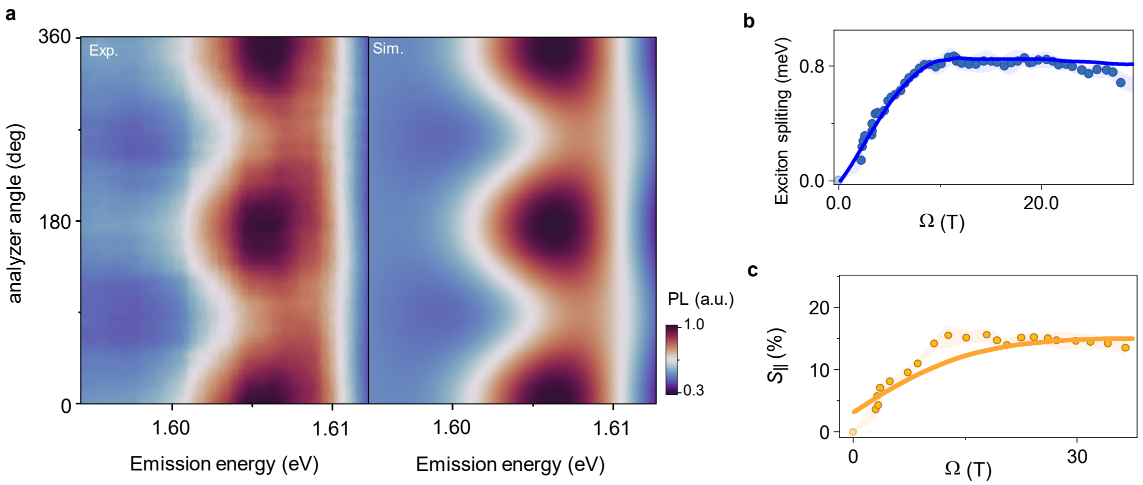

Figure S12: **Zeeman splitting at high pseudomagnetic field a:** Experimentally measured and simulated false-color map of neutral exciton photoluminescence in $MoSe_2$ as a function of analyzer angle under a pseudomagnetic field of 21 T normalized to global maximum. Despite the underlying Zeeman splitting of approximately 2.5 meV, the splitting extracted using the procedure described in the main text yields approximately 0.8 meV in both experimental and simulated maps. **b:** Extracted exciton splitting versus pseudomagnetic field for experimental data (points) and independent simulation (solid line). **c:** Parallel component of pseudospin $S_{||}$ versus pseudomagnetic field for experimental data (points) and independent simulation (solid line).

References

[1] Ivchenko, E. L. *Optical spectroscopy of semiconductor nanostructures* (Alpha Science, Harrow UK, 2005).

[2] Glazov, M. M. *et al.* Spin and valley dynamics of excitons in transition metal dichalcogenide monolayers. *physica status solidi (b)* **252**, 2349–2362 (2015). URL https://onlinelibrary.wiley.com/doi/abs/10.1002/pssb.201552211. _eprint: https://onlinelibrary.wiley.com/doi/pdf/10.1002/pssb.201552211.

[3] Glazov, M. M. Coherent spin dynamics of excitons in strained monolayer semiconductors. *Phys. Rev. B* **106**, 235313 (2022). URL https://link.aps.org/doi/10.1103/PhysRevB.106.235313.

[4] Yu, H., Liu, G. B., Gong, P., Xu, X. & Yao, W. Dirac cones and dirac saddle points of bright excitons in monolayer transition metal dichalcogenides. *Nat Commun* **5**, 1–7 (2014). URL http://dx.doi.org/10.1038/ncomms4876. Publisher: Nature Publishing Group.

[5] Glazov, M. M. *et al.* Exciton fine structure and spin decoherence in monolayers of transition metal dichalcogenides. *Phys. Rev. B* **89**, 201302 (2014). URL https://link.aps.org/doi/10.1103/PhysRevB.89.201302.

[6] Glazov, M. M. *et al.* Exciton fine structure splitting and linearly polarized emission in strained transition-metal dichalcogenide monolayers. *Phys. Rev. B* **106**, 125303 (2022). URL http://arxiv.org/abs/2206.13847. 2206.13847[cond-mat].

[7] Glazov, M. M. Magnetic field effects on spin relaxation in heterostructures. *Phys. Rev. B* **70**, 195314 (2004). URL http://link.aps.org/abstract/PRB/v70/e195314.

[8] Maialle, M., de Andrada e Silva, E. & Sham, L. Exciton spin dynamics in quantum wells. *Phys. Rev. B* **47**, 15776 (1993). URL https://doi.org/10.1103/PhysRevB.47.15776.

[9] Gantmakher, V. F. & Levinson, Y. B. *Carrier Scattering in Metals and Semiconductors* (North-Holland Publishing Company, 1987).

[10] Landau, L. & Lifshitz, E. *Statistical Physics, Part 1* (Butterworth-Heinemann, Oxford, 2000).

[11] Glazov, M. *Electron & Nuclear Spin Dynamics in Semiconductor Nanostructures*. Series on Semiconductor Science and Technology (OUP Oxford, 2018).

[12] Deych, L. I., Erementchouk, M. V., Lisyansky, A. A., Ivchenko, E. L. & Voronov, M. M. Exciton luminescence in one-dimensional resonant photonic crystals: A phenomenological approach. *Phys. Rev. B* **76**, 075350 (2007).

[13] Averkiev, N. S., Glazov, M. M. & Poddubnyi, A. N. Collective modes of quantum dot ensembles in microcavities. *JETP* **108**, 836 (2009).

[14] Mei, T., Lee, J., Xu, Y. & Feng, P. X. Frequency tuning of graphene nanoelectromechanical resonators via electrostatic gating. *Micromachines* **9**, 312 (2018). URL https://www.mdpi.com/2072-666X/9/6/312.

[15] Lloyd, D. *et al.* Band Gap Engineering with Ultralarge Biaxial Strains in Suspended Monolayer MoS_2. *Nano Letters* **16** (2016). URL `https://pubs.acs.org/doi/10.1021/acs.nanolett.6b02615`.

[16] Frisenda, R. *et al.* Biaxial strain tuning of the optical properties of single-layer transition metal dichalcogenides. *npj 2D Materials and Applications* **1** (2017).

[17] Carrascoso, F., Li, H., Frisenda, R. & Castellanos-Gomez, A. Strain engineering in single-, bi-and tri-layer MoS_2, $MoSe_2$, WS_2 and WSe_2. *Nano Research* **14**, 1698–1703 (2021).

[18] Nam, D., Lee, J.-U. & Cheong, H. Excitation energy dependent Raman spectrum of $MoSe_2$. *Scientific Reports* **5**, 17113 (2015).

[19] Tonndorf, P. *et al.* Photoluminescence emission and Raman response of monolayer MoS_2, $MoSe_2$, and WSe_2. *Optics Express* **21**, 4908 (2013).

[20] Lee, J.-U. *et al.* Strain-shear coupling in bilayer MoS_2. *Nature Communications* **8**, 1370 (2017).

[21] Çakıroğlu, O., Island, J. O., Xie, Y., Frisenda, R. & Castellanos-Gomez, A. An Automated System for Strain Engineering and Straintronics of 2D Materials. *Advanced Materials Technologies* **8**, 2201091 (2023).

[22] Doratotaj, D., Simpson, J. R. & Yan, J.-A. Probing the uniaxial strains in MoS_2 using polarized Raman spectroscopy: A first-principles study. *Physical Review B* **93**, 075401 (2016).

[23] Tornatzky, H., Kaulitz, A.-M. & Maultzsch, J. Resonance profiles of valley polarization in single-layer MoS_2 and $MoSe_2$. *Physical Review Letters* **121**, 167401 (2018). URL `https://link.aps.org/doi/10.1103/PhysRevLett.121.167401`.

[24] Baranowski, M. *et al.* Dark excitons and the elusive valley polarization in transition metal dichalcogenides. *2D Materials* **4**, 025016 (2017). URL `https://iopscience.iop.org/article/10.1088/2053-1583/aa58a0`.

[25] Kourmoulakis, G. *et al.* Biaxial strain tuning of exciton energy and polarization in monolayer WS_2. *Applied Physics Letters* **123**, 223103 (2023). URL `https://doi.org/10.1063/5.0167724`.

[26] Boule, C. *et al.* Coherent dynamics and mapping of excitons in single-layer $MoSe_2$ and WSe_2 at the homogeneous limit. *Physical Review Materials* **4**, 034001 (2020). URL `https://link.aps.org/doi/10.1103/PhysRevMaterials.4.034001`.

[27] Dufferwiel, S. *et al.* Valley coherent exciton-polaritons in a monolayer semiconductor. *Nature Communications* **9**, 4797 (2018). URL `https://www.nature.com/articles/s41467-018-07249-z`. Publisher: Nature Publishing Group.

[28] Madéo, J. *et al.* Directly visualizing the momentum-forbidden dark excitons and their dynamics in atomically thin semiconductors. *Science* **370**, 1199–1204 (2020). URL `https://www.science.org/doi/10.1126/science.aba1029`.

[29] Bange, J. P. *et al.* Ultrafast dynamics of bright and dark excitons in monolayer WSe_2 and heterobilayer WSe_2/MoS_2. *2D Materials* **10**, 035039. URL `https://dx.doi.org/10.1088/2053-1583/ace067`.

[30] Godde, T. *et al.* Exciton and trion dynamics in atomically thin $MoSe_2$ and WSe_2 : Effect of localization. *Physical Review B* **94**, 165301. URL https://link.aps.org/doi/10.1103/PhysRevB.94.165301.

[31] Chow, C. M. *et al.* Phonon-assisted oscillatory exciton dynamics in monolayer $MoSe_2$. *npj 2D Materials and Applications* **1**, 1–6. URL https://www.nature.com/articles/s41699-017-0035-1.

[32] Wang, G. *et al.* Polarization and time-resolved photoluminescence spectroscopy of excitons in $MoSe_2$ monolayers. *Applied Physics Letters* **106**, 112101. URL https://doi.org/10.1063/1.4916089.

[33] Wagner, K. *et al.* Nonclassical exciton diffusion in monolayer WSe_2. *Physical Review Letters* **127**, 076801 (2021). URL https://link.aps.org/doi/10.1103/PhysRevLett.127.076801.

[34] Xiao, K. *et al.* Hot exciton effect in photoluminescence of monolayer transition metal dichalcogenide. *Natural Sciences* **3**, e20220035 (2023).

[35] Raiber, S. *et al.* Ultrafast pseudospin quantum beats in multilayer WSe_2 and $MoSe_2$. *Nature Communications* **13**, 4997 (2022). URL https://www.nature.com/articles/s41467-022-32534-3. Number: 1 Publisher: Nature Publishing Group.

[36] Lagarde, D. *et al.* Efficient electron spin relaxation by chiral phonons in WSe_2 monolayers. *Physical Review B* **110**, 195403. URL https://link.aps.org/doi/10.1103/PhysRevB.110.195403.

[37] Fang, H. *et al.* Control of the exciton radiative lifetime in van der Waals heterostructures. *Physical review letters* **123**, 067401 (2019).

[38] Glazov, M. M. *et al.* Exciton fine structure and spin decoherence in monolayers of transition metal dichalcogenides. *Phys. Rev. B* **89**, 201302 (2014). URL http://dx.doi.org/10.1103/PhysRevB.89.201302.

[39] Yu, H., Liu, G.-B., Gong, P., Xu, X. & Yao, W. Dirac cones and Dirac saddle points of bright excitons in monolayer transition metal dichalcogenides. *Nat Commun* **5**, 3876 (2014). URL http://dx.doi.org/10.1038/ncomms4876.

[40] Iakovlev, Z. A. & Glazov, M. M. Longitudinal-transverse splitting and fine structure of Fermi polarons in two-dimensional semiconductors. *J. Lumin.* **273**, 120700 (2024). URL https://www.sciencedirect.com/science/article/pii/S0022231324002643.

[41] Prazdnichnykh, A. I. *et al.* Control of the exciton valley dynamics in atomically thin semiconductors by tailoring the environment. *Phys. Rev. B* **103**, 085302 (2021). URL https://link.aps.org/doi/10.1103/PhysRevB.103.085302.

[42] Mark, J. E. Polymer data handbook. *Oxford University Press* (2009).

[43] Selig, M. *et al.* Excitonic linewidth and coherence lifetime in monolayer transition metal dichalcogenides. *Nature Communications* **7**, 13279 (2016). URL http://dx.doi.org/10.1038/ncomms13279.

[44] Shree, S. *et al.* Observation of exciton-phonon coupling in $MoSe_2$ monolayers. *Phys. Rev. B* **98**, 035302 (2018). URL https://link.aps.org/doi/10.1103/PhysRevB.98.035302.

[45] Glazov, M. M. Quantum interference effect on exciton transport in monolayer semiconductors. *Phys. Rev. Lett.* **124**, 166802 (2020). URL https://link.aps.org/doi/10.1103/PhysRevLett.124.166802.

[46] Grimaldi, C. Electron spin dynamics in impure quantum wells for arbitrary spin-orbit coupling. *Phys. Rev. B* **72**, 75307 (2005). URL https://doi.org/10.1103/PhysRevB.72.075307.

[47] Averkiev, N. S. & Glazov, M. M. Specific features of optical orientation and relaxation of electron spins in quantum wells with a large spin splitting. *Semiconductors* **42**, 958–966 (2008). URL https://doi.org/10.1134/S1063782608080149.

[48] Glazov, M. M. Optical properties of charged excitons in two-dimensional semiconductors. *The Journal of Chemical Physics* **153**, 034703 (2020). URL http://arxiv.org/abs/2004.13484. 2004.13484[cond-mat].

[49] Goldstein, T. *et al.* Ground and excited state exciton polarons in monolayer $MoSe_2$. *The Journal of Chemical Physics* **153**, 071101 (2020). URL https://doi.org/10.1063/5.0013092.

[50] Liu, E. *et al.* Exciton-polaron rydberg states in monolayer $MoSe_2$ and WSe_2. *Nature Communications* **12**, 6131 (2021). URL https://www.nature.com/articles/s41467-021-26304-w. Publisher: Nature Publishing Group.

[51] Iakovlev, Z. A. & Glazov, M. M. Fermi polaron fine structure in strained van der waals heterostructures. *2D Materials* **10**, 035034 (2023). URL https://iopscience.iop.org/article/10.1088/2053-1583/acdd81.

[52] Li, J., Jia, L., Zheng, X., Peng, C. & Fu, X. Structural and elastic properties of wse_2: first-principles calculations. *Journal of Physics: Conference Series* **1634**, 012145 (2020). URL https://dx.doi.org/10.1088/1742-6596/1634/1/012145.

[53] Falin, A. *et al.* Mechanical Properties of Atomically Thin Tungsten Dichalcogenides: WS_2, WSe_2, and WTe_2. *ACS Nano* **15**, 2600–2610 (2021). URL https://doi.org/10.1021/acsnano.0c07430.
